# Metabolic Profiling and Flux Analysis of MEL-2 Human Embryonic Stem Cells during Exponential Growth at Physiological and Atmospheric Oxygen Concentrations

**DOI:** 10.1371/journal.pone.0112757

**Published:** 2014-11-20

**Authors:** Jennifer Turner, Lake-Ee Quek, Drew Titmarsh, Jens O. Krömer, Li-Pin Kao, Lars Nielsen, Ernst Wolvetang, Justin Cooper-White

**Affiliations:** 1 Tissue Engineering and Microfluidics Laboratory, The Australian Institute for Bioengineering and Nanotechnology, The University of Queensland, St Lucia, Queensland, Australia; 2 Stem Cell Engineering Group, The Australian Institute for Bioengineering and Nanotechnology, The University of Queensland, St Lucia, Queensland, Australia; 3 Centre for Systems and Synthetic Biotechnology, The Australian Institute for Bioengineering and Nanotechnology, The University of Queensland, St Lucia, Queensland, Australia; 4 Metabolomics Australia, The Australian Institute for Bioengineering and Nanotechnology, The University of Queensland, St Lucia, Queensland, Australia; 5 School of Chemical Engineering, The University of Queensland, St Lucia, Queensland, Australia; University of Melbourne, Australia

## Abstract

As human embryonic stem cells (hESCs) steadily progress towards regenerative medicine applications there is an increasing emphasis on the development of bioreactor platforms that enable expansion of these cells to clinically relevant numbers. Surprisingly little is known about the metabolic requirements of hESCs, precluding the rational design and optimisation of such platforms. In this study, we undertook an in-depth characterisation of MEL-2 hESC metabolic behaviour during the exponential growth phase, combining metabolic profiling and flux analysis tools at physiological (hypoxic) and atmospheric (normoxic) oxygen concentrations. To overcome variability in growth profiles and the problem of closing mass balances in a complex environment, we developed protocols to accurately measure uptake and production rates of metabolites, cell density, growth rate and biomass composition, and designed a metabolic flux analysis model for estimating internal rates. hESCs are commonly considered to be highly glycolytic with inactive or immature mitochondria, however, whilst the results of this study confirmed that glycolysis is indeed highly active, we show that at least in MEL-2 hESC, it is supported by the use of oxidative phosphorylation within the mitochondria utilising carbon sources, such as glutamine to maximise ATP production. Under both conditions, glycolysis was disconnected from the mitochondria with all of the glucose being converted to lactate. No difference in the growth rates of cells cultured under physiological or atmospheric oxygen concentrations was observed nor did this cause differences in fluxes through the majority of the internal metabolic pathways associated with biogenesis. These results suggest that hESCs display the conventional Warburg effect, with high aerobic activity despite high lactate production, challenging the idea of an anaerobic metabolism with low mitochondrial activity. The results of this study provide new insight that can be used in rational bioreactor design and in the development of novel culture media for hESC maintenance and expansion.

## Introduction

The pluripotent nature of human embryonic stem cells (hESCs) along with their capacity for unlimited self-renewal makes them ideal candidates for use in regenerative medicine. However, before this potential can truly be realised expansion of hESCs to clinically relevant numbers must be based on a more detailed understanding of their metabolic and growth characteristics compared with workhorse lines such as CHO cells [1]. This has led to research and exploration into hESC expansion [2], [3], bioreactor platforms [4]–[7] and the development of maintenance media [8]–[13] and has led to an explosion in biological research to understand the molecular mechanisms governing hESC behaviour. There has been very little exploration however into the fundamental metabolic requirements necessary to support cell expansion in a pluripotent state. Such data would enable the rational design of hESC expansion systems. This work, which describes an in-depth study of hESC metabolism during the exponential growth phase, addresses this deficit.

Human ESC cultures are generally considered to be highly metabolically active, with energy substrates such as glucose being rapidly consumed and waste products such as lactate and ammonia rapidly produced [14]. This provides an explanation for why daily medium changes are necessary in routine hESC culture, a constraint highlighted in a recent study that demonstrated that lactate levels of 25 mM or above inhibit proliferation of hESCs [14] and effects the pluripotent state as indicated by a reduction in Tra-1-60 expression [14].

In addition to being highly metabolically active, previous work has suggested that ESCs are also highly glycolytic [14]–[16], with lactate production to glucose consumption ratios reported to be between 1.8 and 2.8 [14], [15] of a theoretical maximum of 2. This indicates that the majority of the pyruvate generated from glucose metabolism is converted to lactate, rather than entering the mitochondria and the citric acid (TCA) cycle. In contrast, adult mammalian cells typically exhibit lactate to glucose ratios of 1.5 to 1.7 [17]. hESCs are also known to have fewer mitochondria than terminally differentiated cells [18], and possess mitochondria that appear immature and lack normal cristae [16], [19]. The highly glycolytic nature of hESCs combined with the immature structure of their mitochondria has led to the proposition that the mitochondria in hESCs are less active than in differentiated cells, and that energy generation by oxidative phosphorylation in the mitochondria plays no or only a minor role in hESCs [16]. It is interesting to note however, that a recent study found that expression of TCA cycle genes is higher in hESCs than in differentiated cells [16].

Since hESCs are isolated from the inner cell mass of a day 5 blastocyst prior to implantation [20] and *in vivo* reside in a hypoxic environment [21], the effect of oxygen tension on hESC behaviour has been an area of intense research. Physiological oxygen concentrations of 1–5% have been shown to improve human and mouse embryonic stem cell ESC survival [22], [23] and enhance the maintenance of pluripotency [24]–[26], when compared with atmospheric oxygen concentrations of 20% typically used in routine hESC maintenance. While there is data available in the literature on amino acid [14], glucose [14], [16], lactate [14], [16] and ammonium [14] uptake and production rates for hESCs cultured at atmospheric oxygen concentrations, to date little has been done to determine how the metabolic requirements differ when cultured at physiologically-relevant oxygen concentrations (e.g. 1–5%). In addition, current studies limit themselves to uptake and production rates of only a few key metabolites and have not investigated the activity of internal metabolic pathways that are known to be highly interconnected.

In order to gain greater understanding of hESC metabolism, we have employed metabolic profiling and flux analysis techniques to investigate the metabolic requirements and to predict the activity of the internal metabolic pathways at both physiological (2%) and atmospheric (20%) oxygen concentrations. The metabolic flux analysis (MFA) model developed herein consists of over 2000 reactions, which should realistically approximate hESC metabolism. Quantitative analysis shows that the model accounts for all major carbon sources, amino acids, metabolic by-products, total DNA content and total protein content measured during the exponential growth phase of hESC culture.

To our knowledge, even though it is currently only available for one hESC line, this is the first detailed metabolic flux analysis of hESCs under varying oxygen conditions, and the resultant data the most in depth for hESC metabolism to date. The results of this study will provide a useful resource for researchers interested in probing and understanding hESC metabolism, improving rational bioreactor design and the development of novel culture media for hESC maintenance and expansion.

## Methods

### Cell culture

The hESCs used within this study were of the MEL-2 hESC line, previously characterised by the International Stem Cell Initiative [27]. All cells used within this study were obtained from the Australian Stem Cell Centre, Queensland Node Core hESC Laboratory (StemCore). In order to accurately enumerate cell number for the metabolic flux analysis, it was necessary to be able to passage hESCs as a single cell suspension. Adapted hESCs for passaging as a single-cell suspension (using TrypLE express (Invitrogen)) were provided by StemCore, following protocols adapted from Costa et al 2008 [28]. Briefly, cells were moved from passaging as pieces (W), to bulk enzymatic passaging (Y), to passaging as a single cell suspension on a mouse embryonic fibroblast (MEF) layer (Y), and finally passaging as a single cell suspension in a feeder-free system (Z). Passage numbers were designated as pW+X+Y+Z. Cells received from StemCore were p18+2+9−13. They were then passaged as a single cell suspension for a further two passages on BD Matrigel hESC-qualified matrix (BD Biosciences), used at 1/8 of the recommended concentration, in mTeSR-1 (StemCell Technologies) prior to use in all experiments.

### Cell sorting, culture initiation and sampling

Cell stocks were harvested as a single cell suspension and sorted into well plates for metabolism experiments. Prior to cell detachment the culture medium was supplemented with 10 µM of small molecule Y-27632 dihydrochloride monohydrate (ROCK inhibitor) (Sigma) and incubated for 2 hours. To detach cells in a single cell suspension for sorting, cells were rinsed twice with warm PBS and treated with TrypLE Express (Invitrogen) for 5 minutes at 37°C. Cells were spun down and resuspended in DMEM F12 (Invitrogen) +1% (v/v) Penicillin/Streptomycin (Invitrogen) +10 µM Y-27632 + propidium iodide (PI).

Cells were sorted with either a Cytopeia Influx or a BD FACS Aria Instrument at the Queensland Brain Institute Flow Cytometry Facility, University of Queensland. Cells were sorted into 6-well plates that had been pre-coated with BD Matrigel hESC-qualified matrix (BD Biosciences), at 1/8 of the recommended concentration at 4°C overnight, and filled with 2 mL mTeSR-1 (StemCell Technologies) supplemented with 1% (v/v) Penicillin/Streptomycin (Invitrogen) and 10 µM Y-27632 (Sigma). 120,000 cells were sorted into each well, resulting in an even distribution of cells. Only viable single cells were sorted into wells utilising doublet exclusion (using forward- and side-scatter height and width parameters) and exclusion of cells staining positive for PI. Once sorted the cells were placed in the appropriate incubator, either a Binder CB160 variable oxygen incubator at 2% O_2_ and 5% CO_2_ or a standard Sanyo CO_2_ incubator set at 20% O_2_ and 5% CO_2_. The cells were left overnight to attach. The cell culture medium was then exchanged with 2 mL fresh mTeSR-1 supplemented with 1% (v/v) Penicillin/Streptomycin (Invitrogen) per well to remove Y-27632 and any unattached cells from the system. Note that media exchange was performed using media preconditioned to match oxygen concentration, that is, either preconditioned hypoxic media or normoxic media. This media change marked the commencement of the experiment and was considered time  = 0 hours.

Samples were then collected approximately every eight hours for 4–5 days. For each biological replicate at each timepoint, the following procedure was conducted: 1) Phase-contrast images were taken using an Olympus IX81 inverted microscope; 2) Cell culture supernatant was collected, filtered with a 0.22 µm syringe filter and frozen at −20°C until analysis; and 3) Cells were harvested using TrypLE express (Invitrogen) and fixed for 10 minutes in filtered ice-cold 70% ethanol and stored in PBS +2% foetal bovine serum (FBS) (Invitrogen) at 4°C until cell number enumeration analysis.

### Cell imaging

Phase contrast images were taken of each biological replicate at time  = 0 hours and each subsequent timepoint. Images were taken using an Olympus IX81 inverted microscope with a 4x objective lens. Pre-programmed stage positions, using Cell-R software, were utilised to ensure that images were taken at the same position within the culture well at each timepoint.

### Cell enumeration by flow cytometry

Flow cytometry was used to quantify cell numbers throughout the study. 35 µL of a known concentration of Flow-Count Fluorospheres (Beckman Coulter) was added to a fixed cell sample and vortexed to mix. Approximately 30 minutes prior to flow cytometry analysis, 10 ug/mL Hoechst 33342 (Invitrogen, Molecular Probes) was added to each sample to obtain a cell cycle profile to allow cells to be readily discriminated from debris. Samples were analysed on a BD LSR II flow cytometer at the Queensland Brain Institute Flow Cytometry Facility. Counting beads were identified with a FITC (530/30) detector, and Hoechst positive cells were identified with the (450/50) detector, see Figure S1 for more detail. Multiplets could be discerned from the Hoechst histogram as cells brighter than the G2/M peak. Events were recorded until 1000 beads had been counted. Cell number could then be determined by Equation 1: Total cells  =  ((volume of beads added x bead concentration)/(#beads counted)) x (# cells counted + # doublets).

### Biochemical analysis

Frozen cell culture supernatant samples were thawed, vortex-mixed and subsequently analysed with a Bioprofile FLEX Chemistry Analyser (Nova Biomedical, Waltham). This analysis provided data for ammonia concentration, pH and osmolality.

### High-Performance Liquid Chromatography

Extracellular metabolite concentrations were analysed by high performance liquid chromatography (HPLC). All samples were deproteinated via ultrafiltration (<3 kDa) prior to analysis. Amino acid analysis was performed as described previously [29], except that cysteine was not quantified. Organic acids and glucose were quantified with UV and RI detection, respectively. Separation of compounds was achieved on a Rezex RHM-monosaccharide column (300×7.8 mm, 8 µm, Phenomenex) at 70°C and 0.6 mL min^−1^ of 4 mM H_2_SO_4_ in water.

### Determining total cellular protein content

Cells were sorted into 6-well plates as described above. Cells were cultured at 20% oxygen, as it was assumed that oxygen concentration does not affect total protein. Cells were harvested at timepoints approximately 40 hours, 70 hours and 100 hours after the initial media exchange. At each timepoint cells were harvested with TrypLE express (Invitrogen) and then resuspended in 1 mL of DMEM-F12 (Invitrogen). A known number of viable single cells, either 1×10^5^ or 2×10^5^ cells, were then sorted into a tube using a Cytopeia Influx cell sorter as described above. Once sorted, the cells were spun down and a known volume of liquid supernatant removed. This was then replaced with 150 µL of RIPA buffer (100 mM EDTA, 50 mM Tris-HCL, pH 8, 150 mM NaCl, 1% Triton, 0.5% sodium deoxycholate, 0.1% SDS) + 1 x complete EDTA-free protease inhibitor cocktail (ROCHE Applied Science). The tube weight was measured before and after sorting, this combined with controlling the volume of liquid in the tube allowed the cell number per mL to be calculated with a high degree of accuracy. This step was critical to ensuring that our cell numbers were consistent throughout all experiments, allowing for closure of the flux analysis. The protein concentration was determined using a BCA protein assay kit (Pierce Thermo Scientific) according to the manufacturer's instructions. All samples and standards were diluted with a mixture of PBS and RIPA buffer to a comparable concentration. The plate was read on a Spectromax plate reader. Finally, the protein concentration per cell was determined by dividing the protein concentration by the total number of cells in the sample. Three biological replicates were included per experiment and the experiment repeated twice.

### Determining total DNA content

Cellular DNA content was ascertained by adding 10 ug/mL Hoechst 33342 (Invitrogen, Molecular Probes) to samples taken at various timepoints for cell enumeration. Staining was detected using a BD LSRII flow cytometer equipped with a UV laser and 450/50 nm detector. Flow cytometric data were analysed in WEASEL v2.7.4 software (Walter and Eliza Hall Institute for Medical Research, Melbourne, Australia). Multiplets were excluded from analysis by gating on forward- and side-scatter area, height, and width parameters. Cells in different stages of the cell cycle (G0/G1, S, G2/M) were estimated by using the Curve Fit - Cell Cycle function in Weasel. This function fits Gaussian distributions to the G0/G1 and G2/M peaks identified in the DNA histogram, and calculates percentages of cells in each compartment. Samples to which curves could not be fitted were discarded. Cell cycle profiles were determined for multiple (n = 4–6) biological replicates at each timepoint.

### Metabolic Flux Analysis

The intracellular fluxes of hESC cultures were determined by linear programming using the measured extracellular metabolite concentrations and cell numbers as constraints [30]. The mouse genome scale model was used because it adequately represents core mammalian metabolism and can be directly applied to cell culture flux experiments [31]. Briefly, intracellular fluxes (***v***) can be calculated using the metabolite balancing constraints ***S***•***v***  = 0, whereby ***S*** is the stoichiometric matrix derived from the metabolic model, and that cell metabolism is assumed to be at a pseudo-steady state. The constraint v ≥0 is imposed on all irreversible reactions, while the lower and upper boundary values of measured fluxes were specified using the measured cell-specific consumption or production rates and the estimated standard error (v_measured_ ± SE_measured_). The maximum ATP yield objective function is used in order to generate a version of flux distributions that is energetically most efficient. Flux calculations were done in MATLAB (The Mathworks) using a third-party LP solver (Gurobi Optimizer and Gurobi Mex).

The biomass composition of hESC was approximated using literature values of hybridoma cell lines. It was assumed that RNA content is 3 times of DNA, and that lipid and carbohydrate contents are 1/7 and 1/10 of protein, respectively [32]. The relative fractions of amino acids, nucleotides and lipids within each subgroup were also used (Sheikh 2005). The biomass composition was further refined using measured cellular protein and DNA content of hESC. This was accomplished by adjusting the absolute amount (mmol per cell) of each biomass component such that the weights of the total protein, DNA, RNA, lipid and carbohydrate are met, while maintaining the same relative fractions of amino acids, nucleotides and lipids within each subgroup.

### Blocking mitochondrial complex I and II

MEL2 hESCs were seeded at 200,000 cells per well in a 6-well plate coated with BD Matrigel hESC qualified matrix (BD Biosciences). Cells were cultured for 60 hours to ensure that they were in the exponential phase of growth. Cell culture medium was exchanged with either fresh 2 mL of mTeSR-1 (StemCell Technologies) or mTeSR-1 supplemented with 50 µM α-tocopherol succinate (α-TOS) (Sigma) to block mitochondrial respiratory complex II [33], or 0.5 µg/mL rotenone (Sigma) to block mitochondrial complex I [34], or 0.01% ethanol as a carrier control for α-TOS, or 0.01% DMSO as a carrier control for rotenone. The cells were incubated for a further 24 hours prior to harvesting with TrypLE express (Invitrogen) and cell enumeration. All experiments were performed in triplicate.

### Western blot analysis

For HIF-1α protein quantification cells were lysed at 80 hours during the exponential growth phase in RIPA buffer (150 mM sodium chloride, 1% Triton X−100, 1% sodium deoxycholate, 0.1% SDS, 50 mM Tris-HCl, pH 7.5, and 2 mM EDTA) supplemented with 1x EDTA-free protease inhibitor cocktail (Roche Applied Science), as recommended by the manufacturer. Cell lysates were then loaded to 6% polyacrylamide gel and electrotransferred onto nitrocellulose membranes. Membranes were blocked with 5% skim milk in TBST (10 mM Tris-HCl buffer, pH 7.6, 150 mM NaCl, and 0.1% Tween-20) for 1 h. Membranes were then incubated with a primary antibody against HIF1α (610958, BD Transduction Laboratories) diluted 1∶1000 in TBST and β-tubulin antibody (088K4795, Sigma) diluted 1∶10000 in TBST, respectively at 4°C overnight. Primary antibody was detected with a horseradish peroxidase-conjugated anti-mouse secondary antibody (12–348 and 12–349, Millipore) following a 1 hour incubation at RT using the ECL Western blotting detection system (GE Healthcare). Moreover, we detected two bands, the expected ∼120 kDa band for HIF1α, and a smaller size band of protein of ∼80 kDa molecular weight, corresponding to a lower molecular weight splice variant of HIF1α that has previously been reported in human cells. For the purposes of analysis and comparison, the relative HIF1-α protein concentrations were determined from the sum of both bands using Image J following protocols described in the literature [35] and normalised to β-tubulin protein expression determined using the same protocol. The apparent molecular weights of the proteins detected were 120, 80 and 55 kDa, for HIF1α, HIF1α splice variant (sv) and β-tubulin, respectively. Three biological replicates were measured at both 2% and 20% oxygen.

### Quantitative real-time PCR (q-PCR)

Total RNA was extracted from cells at 80 hours during the exponential growth phase at 2% and 20% oxygen with a Qiagen RNeasy Mini RNA extraction kit (Qiagen) and quantified with a NanoDrop 1000 spectrophotometer. cDNA was synthesized, using 1.5 µg of total RNA and 1 µl d(T)_20_ (500 µg/ml) (Geneworks) mixed with RNase-free water to a final volume of 12 µl. The reaction was incubated at 65°C for 5 min, placed on ice, and then 4 µl 5× first-strand buffer and 2 µl 0.1 M DTT (Invitrogen) were added and incubated at 37°C for 2 min. Finally, 1 µl (200 U) MMLV reverse transcriptase (Invitrogen) was added to the reaction and incubated at 37°C for 50 min, followed by incubation at 70°C for 15 min. The resulting cDNA was used for quantitative real-time PCR (qPCR) analysis.

Expression of the genes, HIF1-α, HIF2-α, and β-actin were quantified by qPCR using an ABI 7500 detection system (Applied Biosystems), with fluorescein as an internal passive reference dye for normalization of well-to-well optical variation. PCR amplification was performed in a total volume of 10 µl containing 5 µl 2x SYBR Green supermix (Applied Biosystems), 0.2 µl primers (10 µM each), 0.2 µl cDNA and DNase-free water (Invitrogen, Gibco). The reaction conditions were as follows: 95°C for 1 min, followed by 40 cycles of 95°C for 30 sec, and 52°C for 30 sec, with a final dissociation step to generate a melting curve for verification of amplification product specificity. Real-time qPCR was monitored and analysed with ABI 7500 fast optical system software. The primers used are as follows; HIF-1α, F: 5′-GTAGTTGTGGAAGTTTATGCTAATATTGTGT-3′, R: 5′-CTTGTTTACAGTCTGCTCA-AAATATCTT-3′; β-actin F: 5′-GCTGTGCTACGTCGCCCTG-3′, R 5′- GGAGGAGCTGGAAGCAGCC-3′. Each reaction was performed in triplicate, and amplification in the presence of a single primer was performed as a negative control. Relative mRNA levels were calculated using the comparative CT method according to the Applied Biosystems manual and normalized to β-actin mRNA. The fold change in expression of each target mRNA relative to β-actin was calculated by the 2-Δ(ΔCT) method [36], [37]. To calculate PCR efficiency, standard curves were generated with serial dilutions of cDNA from experiments performed in triplicate, enabling the determination of CT values and PCR efficiencies for individual assays and variations between individual assays. The PCR efficiency (E) was calculated using the equation E =  (10 [1/−slope]−1)×100). Thus, E is between 110% and 90% when the slope falls between −3.1 and −3.6. The slope was calculated by plotting the fold-dilution of cDNA versus the CT value [38].

### Flow cytometry analysis of pluripotency marker expression

Cell samples at time  = 0 and the endpoint of an experiment were analysed by flow cytometry for expression of pluripotent markers. Samples were blocked with 3% bovine serum albumin (BSA) (Sigma-Aldrich). Samples were incubated for 30 mins at RT with 100 µL of primary antibody against TG30 (1∶400) (MAB4427, Millipore), TRA-1-60 (1∶400) (MAB4360, Millipore) or Oct-4 (1∶400) (MAB4401, Millipore). Samples were washed twice with 500 µL PBS, then incubated for 30 mins at RT with 100 µL of AlexaFluor 488-conjugated secondary antibodies against mouse IgG (H+L) or mouse IgM (µ chain) (1∶500) (Invitrogen). 30 minutes prior to analysis 10 ug/mL Hoechst 33342 (Invitrogen, Molecular Probes) was added to each sample to aid in gating the cell population. Multiplets and debris were excluded from the marker expression analysis, as described above.

### Karyotype analysis

Regular karyotype analysis of hESC lines is performed by StemCore as part of their routine quality procedures. The MEL-2 hESCs used within this study were karyotyped at p18+2+6 and p18+2+15.

## Results

The characterisation of the metabolism of MEL-2 human embryonic stem cells involved three stages. First, the cell growth profile, cell DNA and protein content, as well as the uptake and production rates of key metabolites were measured by experimentation. The second stage utilised the experimental data to develop a fluxomic model of hESC metabolism capable of resolving fluxes through internal metabolic pathways. The third stage involved a preliminary analysis of the results generated by the model to highlight key features of hESC metabolism. All stages of the study were conducted at physiological (2%) and atmospheric (20%) oxygen concentrations for comparison.

### Pluripotency, control of cell culture conditions and cell DNA and protein measurements

**Figure 1:**
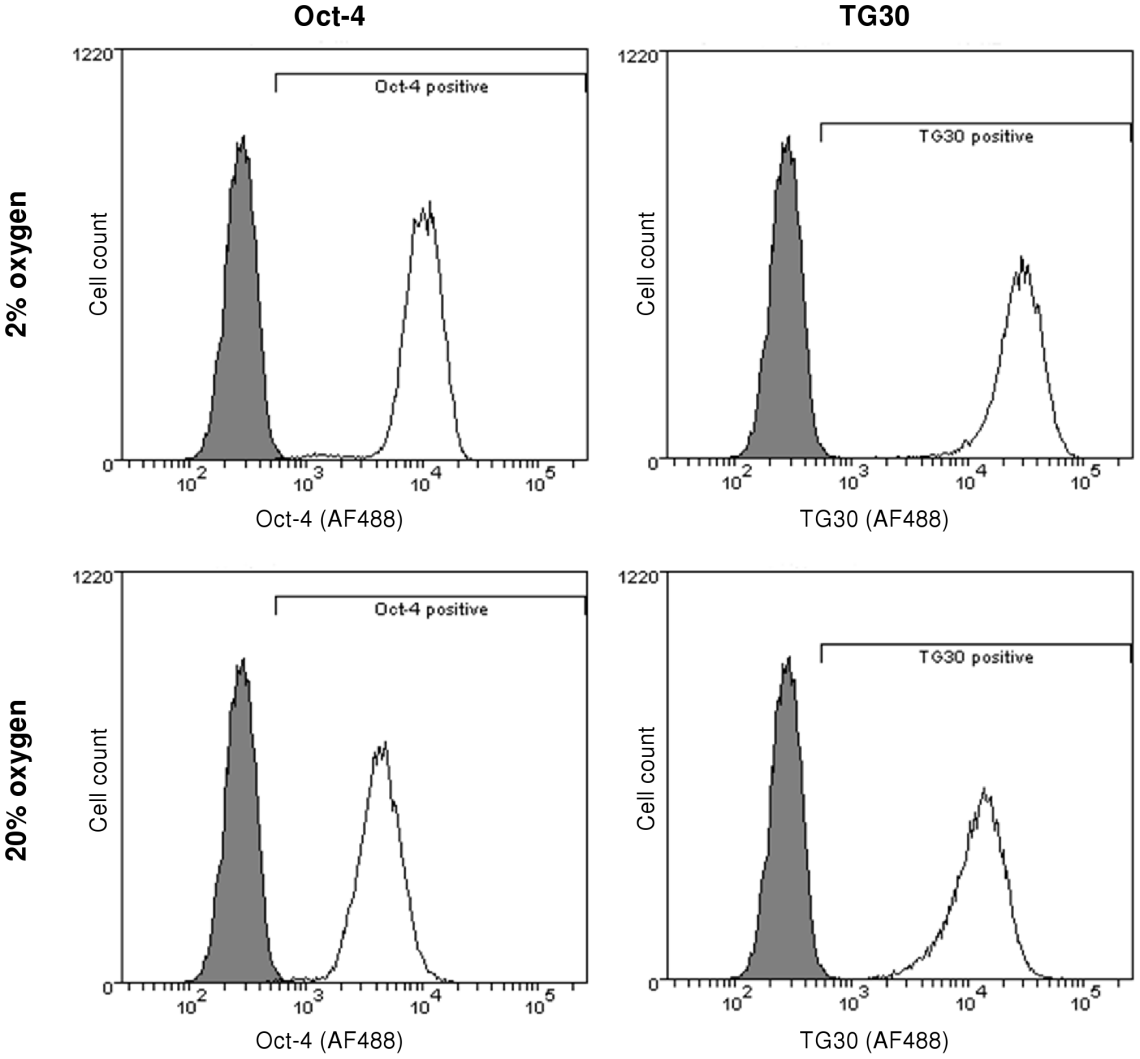
Pluripotent marker expression of human embryonic stem cells. Pluripotent marker expression of hESCs cultured under physiological, 2%, and atmospheric, 20%, oxygen concentrations were tested by FACS analysis after the completion of each experiment to ensure that the cells remained pluripotent throughout the 100 hour experiment. Representative FACS plots showing the shift in marker expression from the negative controls at the endpoint of an experiment are shown. All cell samples were found to be 92–99% positive for both TG30 and Oct-4 after each experiment at both 2% and 20% oxygen.

The experiments were performed with MEL-2 hESCs of passage 2 in a single cell adapted state. Karyotype analysis of the cell stocks was performed at passage 15 in a single cell adapted state. No karyotype abnormalities were detected (Figure S2). The cells were stained for pluripotency markers, Oct-4 and TG30, and analysed by flow cytometry before and after each experiment (Figure 1). Cells were found to be 92–99% positive for both Oct-4 and TG30 after each experiment at both 2% and 20% oxygen.

The pH and osmolality of the cell culture medium supernatant was measured at each time point throughout the experiment (Figure S3). The pH and osmolality were within limits to promote normal cell growth, as defined in Ludwig et al. (2006) [8], throughout the exponential growth phase.

The total protein content of hESCs was measured to be 156±31.9 pg/cell. The total DNA content was estimated from cell cycle data to be 9.39±0.78 pg/cell. These parameters were used to estimate other cell parameters, such as RNA content, using rules of thumb outlined by Bonarius et al. 1996 [32]. See materials and methods for a full list of assumptions.

### hESC exponential growth profiles are similar at physiological and atmospheric O_2_ concentrations

**Figure 2:**
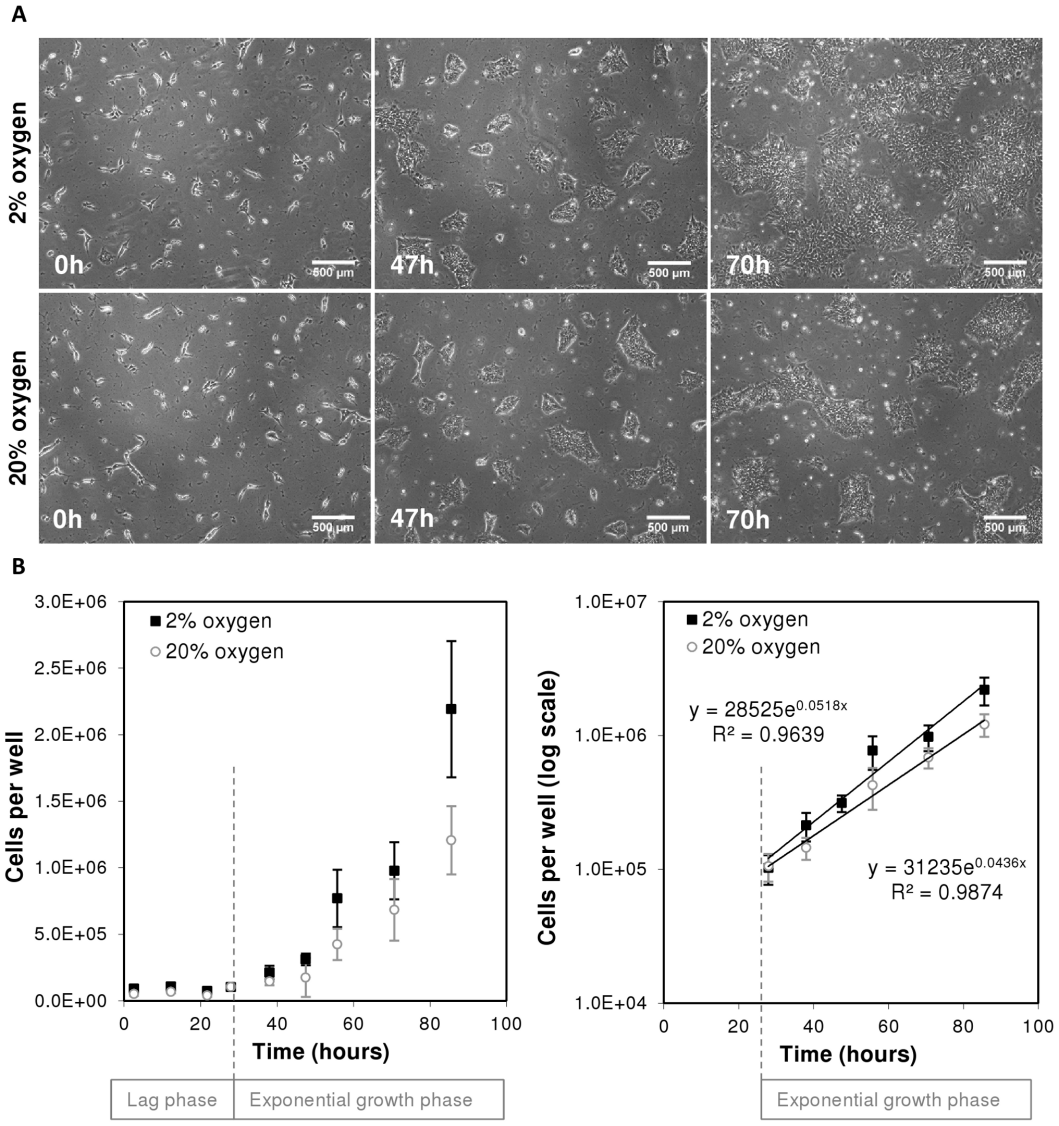
Growth profile of human embryonic stem cells cultured at physiological and atmospheric oxygen concentrations. hESCs were harvested as a single cell suspension and then FACS sorted into 6-well plates to ensure a uniform seeding distribution across all wells. The cells were then cultured at physiological, 2%, and atmospheric, 20%, oxygen concentrations. At each time point cells were harvested and counted using flow cytometry techniques. A) The phase contrast images represent the spatial distribution of cells in the well and colony morphology at time equals 0, 47 and 70 hours after the experiment initialisation. The cell seeding methodologies employed allowed for consistent uniform seeding which translated to consistent initial cell numbers. Scale bar represents 500 µm. B) Growth profile of hESCs showing the lag and exponential growth phase on the semi-log plot to determine the specific growth rates. Values are means ± standard deviation, n = 6.

Throughout this study MEL-2 hESCs were passaged as a single cell suspension using TrypLE, instead of the more traditional enzymatic or manual culture techniques which typically transfer hESCs in clumps of cells to a new culture vessel. Our seeding protocol resulted in a greater control over cell number and allowed for a consistently uniform initial seeding density, which in turn ensured uniform progression through the phases of cell growth within a specific culture condition (Figure 2). The initial lag phase lasted approximately 25 hours after the commencement of the experiment, and was observed to be independent of oxygen concentration (Figure 2). The following exponential growth phase was used to calculate the growth rates. The growth rate was found to be 0.051±0.0025 h^−1^ and 0.043±0.0024 h^−1^ (values ± SE) at 2% and 20% oxygen, respectively (Figure 2). The growth rates were not significantly different, p-value 0.05. Only data from the exponential growth phase was used in constructing the metabolism profile and flux analysis.

### Differences in metabolite uptake and production rates seen with differing O_2_ concentrations

Metabolite concentrations in the cell culture supernatant were measured at each experimental time point to generate concentration profiles (See Figure S4). The concentration profiles were then used in conjunction with cell numbers to determine the specific uptake and production rates (Table 1). Glucose was consumed and lactate produced throughout the culture at 2% and 20% oxygen. The glucose and lactate consumption and production rates were calculated and found to be statistically different at 2% and 20% oxygen, with rates 1.8 times higher at 2% oxygen (Table 1). Despite the difference in rates, the lactate production to glucose consumption ratios (Y_Lac/Glc_) were calculated to be 2.3, at both oxygen concentrations during the exponential phase of growth (Table 2). In contrast to glucose and lactate, ammonia rates were found to be 1.6 times higher at 20% oxygen than at 2% oxygen (Table 1).

**Table 1:** Flux of major metabolites consumed and produced by human embryonic stem cells cultured at physiological and atmospheric oxygen concentrations.

Consumed metabolites	Specific Rates (mmol h^−1^ 112 cells^−1^)		Produced metabolites	Specific Rates (mmol h^−1^ 112 cells^−1^)	
	2% Oxygen	20% Oxygen		2% Oxygen	20% Oxygen
Glucose**	569.91±56.85	307.45±23.03	Lactate**	1316.94±121	712.94±56.62
Aspartate*	2.54±0.56	0.57±0.43	Ammonia**	20.51±1.56	35.32±3.84
Asparagine*	3.27±0.39	1.69±0.40	Glutamate	5.84±0.71	4.63±0.59
Serine	17.92±1.51	12.89±1.18	Alanine**	25.20±2.52	8.20±0.81
Glutamine**	15.62±6.02	68.12±12.68			
Histidine	2.00±0.32	1.79±0.35			
Glycine*	3.82±0.74	0.73±0.76			
Threonine	5.08±0.92	4.18±1.02			
Arginine	26.84±2.33	19.38±2.09			
Tyrosine	2.79±0.48	2.54±0.52			
Valine	7.27±1.03	6.10±1.09			
Methionine	2.83±0.30	2.33±0.30			
Tryptophan	0.91±0.14	1.00±0.14			
Phenylalanine	3.50±0.50	3.06±0.54			
Isoleucine	9.02±1.03	6.77±1.03			
Leucine	10.59±1.18	8.08±1.15			
Lysine	7.55±1.23	4.92±1.18			
Proline	−3.31±1.14	−0.23±0.71			

Note: All data measured by HPLC analysis, except for ammonia, which was measured using the Bioflex analyser. Values are mean ± standard error, n = 6. Significance between the values measured at 20% and 2% oxygen were determined using a student's t-test where ** indicates a p-value<0.05, deemed statistically significant and *indicates a p-value<0.08, deemed statistically significant.

**Table 2:** Biological ratios of human embryonic stem cells cultured at physiological and atmospheric oxygen concentrations.

Biological ratios *	2% Oxygen	20% Oxygen
Lactate production to glucose consumption ratio (Y_Lac/Glc_)	2.31±0.31	2.32±0.25
Ammonia production to glutamine consumption ratio (Y_NH3/Gln_)	1.31±0.52	0.52±0.11
Glucose to glutamine consumption ratio (Y_Glc/Gln_)	36.49±14.53	4.51±0.91
Respiratory quotient (RQ = Y_CO2/O2_)	1.04±0.01	1.09±0.01
Ratio cytoplasmic ATP production to ATP production in the mitochondria	0.83±0.09	0.42±0.06

* Note: The specific consumption and production rates used to calculate the biological ratios for lactate, glucose, ammonia and glutamine were measured while the specific rates for carbon dioxide, oxygen and ATP were calculated from the metabolic flux analysis results.

The amino acid (AA) profile, in terms of the AA′s produced and consumed, was the same regardless of oxygen concentration. There were however some statistically significant differences in the rates. Alanine was produced, and aspartate, asparagine and glycine were consumed at statistically higher rates at 2% oxygen than at 20% oxygen (Table 1). Glutamine, however, was consumed at a statistically higher rate at 20% oxygen compared with 2% oxygen (Table 1). While Y_Lac/Glc_ was the same at both oxygen concentrations, Y_Amm/Gln_ (ammonia production to glutamine consumption ratio) was determined to be 1.31 and 0.52 at 2% and 20% oxygen respectively (Table 2). No AA′s were found to be limiting during the culture.

### Metabolic Flux Analysis shows a difference in energy pathways at physiological and atmospheric O_2_ concentrations

The metabolic flux analysis (MFA) model used within this study consisted of over 2000 internal metabolic reactions. The measured data detailed above was used as inputs to the model to provide constraints on the theoretical solution space, allowing for the mass balance to be closed and a solution to be generated. The output of this model shows that of the 2000 reactions, 288 are considered to be essential for hESC metabolism (Table S1). Of the reactions considered essential, 61 reactions show a significant difference in the flux between hESCs cultured at physiological, 2%, and atmospheric, 20%, oxygen concentrations, with the flux being greater at physiological oxygen in 70% of these reactions (Table S1). At first glance, this suggests that hESCs are more metabolically active at physiological oxygen concentrations compared with atmospheric oxygen concentrations.

Interestingly, overall the fluxes through pathways involved in biogenesis are not statistically different between the two oxygen concentrations (Table S1). This is in keeping with the observation that the growth rates of MEL-2 hESCs at these two oxygen concentrations were not statistically different.

**Figure 3:**
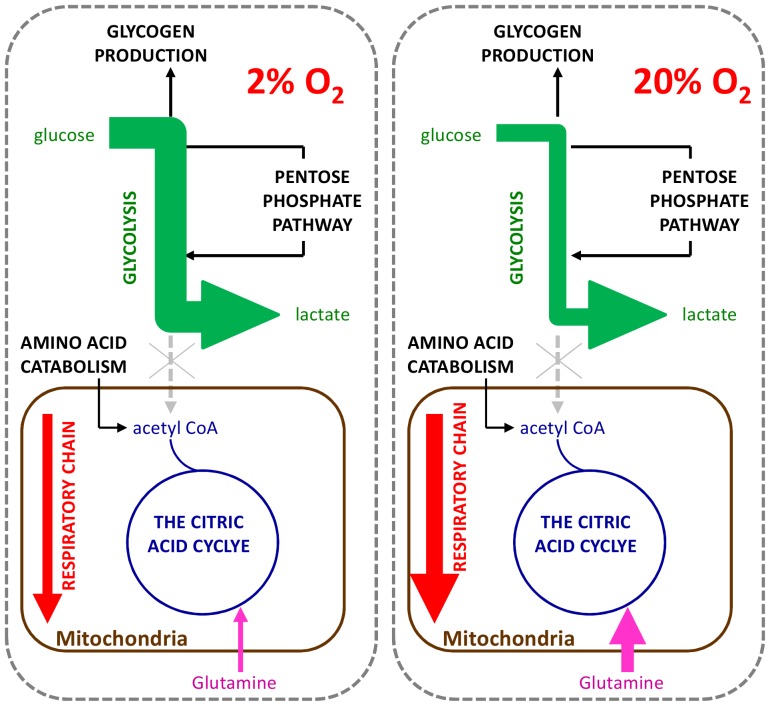
Activity of human embryonic stem cells metabolic pathways cultured at physiological and atmospheric oxygen concentrations. A visual representation of the flux through key metabolic pathways of hESCs cultured at both physiological and atmospheric oxygen concentrations. At both oxygen concentrations glycolysis is disconnected from the TCA cycle. The flux through the glycolysis pathways is greater at physiological oxygen concentrations while the uptake of glutamine and the flux through the respiratory chain is greater at atmospheric oxygen concentrations.

Another similarity is that the results of the model show no statistically significant difference in the total amount of ATP produced by MEL-2 hESCs at physiological and atmospheric oxygen (see Table 3). There is however a significant difference in the flux through the reactions used to generate ATP (see Figure 3).

**Table 3:** ATP production.

	Total flux ATP produced	Total flux ATP produced by Oxidative phosphorylation	Total flux ATP produced by Glycolysis
2% oxygen	219–251 mmol/10^6^ cells	116–141 mmol/10^6^ cells * (53–56% total ATP production)	103–110 mmol/10^6^ cells * (47–44% total ATP production)
20% oxygen	207–252 mmol/10^6^ cells	144–178 mmol/10^6^ cells * (69–71% total ATP production)	64–74 mmol/10^6^ cells * (31–29% total ATP production)

*Note: The range of values reported for ATP flux were calculated from the upper and lower bounds of the resulting fluxes from the MFA as reported in Table S1.

**Figure 4:**
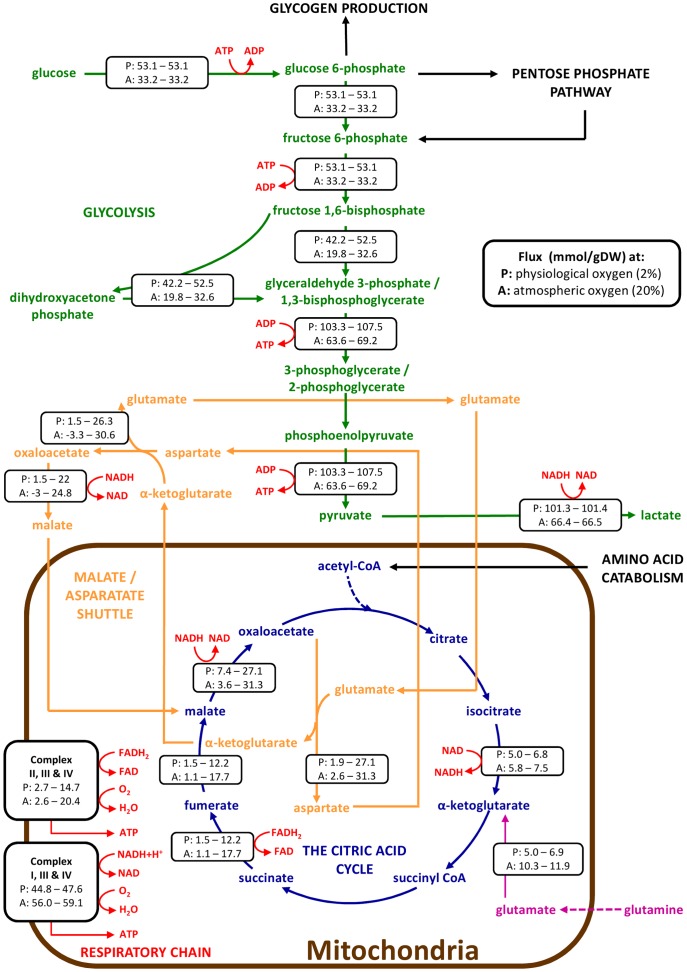
Metabolic pathway fluxes of human embryonic stem cells cultured at physiological and atmospheric oxygen concentrations. Overview of the fluxes through key metabolic pathways of hESCs cultured at physiological, 2%, and atmospheric, 20%, oxygen concentrations. At both oxygen concentrations glycolysis is disconnected from the citric acid cycle with majority of the glucose being converted to lactate, with some being diverted to glycogen production and the pentose phosphate pathway. The mitochondria is metabolically active with glutamine serving as the main carbon source for ATP aerobic respiration. The TCA cycle within the mitochondria is driven by the catabolism of amino acids. Values represent the minimum and maximum flux through the pathway at both physiological (P) and atmospheric (A) oxygen concentrations as determined by the MFA model developed within this study.

At physiological oxygen concentrations, the flux through the glycolysis pathway is much greater than that at atmospheric oxygen concentrations (Figures 3 and 4). It is interesting to note however, that unlike most terminally differentiated cells, in MEL-2 hESCs, glycolysis is completely disconnected from the citric acid (TCA) cycle, with all of the pyruvate being converted to lactate (Figures 3 and 4). This phenomenon is observed at both oxygen concentrations, and further, is supported by the high Y_Lac/Glc_ measured (see Table 2).

This MFA model however shows that the mitochondria and TCA cycle are still active and facilitates the catabolism of amino acids. In fact the TCA cycle is thus mostly driven by catabolism of amino acids, and there is no net oxidation of glucose carbon into CO2 via the TCA cycle. In addition, glutamine is fed into the TCA cycle, following transamination to α-ketoglutarate, acting as a carbon source for ATP production by oxidative phosphorylation (OXPHOS) (Figure 4). Consumption of glutamine dominated in the 20% cells, while consumption of arginine, glutamine and serine had comparable contributions in the 2% cells. In the 20% cells, the catabolism of glutamine accounted for up to 79% of the oxygen consumed (Calculated from data given in Table S1). For the 2% cells, glutamine catabolism required at most 28% of the total oxygen consumed. For the same amount of ATP produced, 20% cells needs 3 times more O2 (Calculated from data given in Table S1), i.e., TCA cycle activity is 3 times greater. Without oxygen, 2% cells can make 56 mmol/gDW/hr ATP, while 20% cells can make 17 mmol/gDW/hr ATP (Calculated from data given in Table S1).

The ratio of cytoplasmic to mitochondrial ATP production of hESCs is twice as high at physiological oxygen compared to atmospheric oxygen (see Table 2). This is driven by a glucose to glutamine consumption ratio, Y_Glc/Gln_, that is eight times higher at physiological oxygen compared with atmospheric (see Table 2).

### Oxygen uptake

The oxygen uptake rate, OUR, of MEL-2 hESCs was calculated from the results of the MFA to be 369–375 mmol h^−1^ 10^12^ cells^−1^ when cultured at 2% oxygen and 397–403 mmol h^−1^ 10^12^ cells^−1^ when cultured at 20% oxygen. The oxygen uptake rates (OUR) were used to determine the maximum number of cells that could be supported in this experimental set-up before oxygen became limiting using Equation 2 (Equation 2: Total cells  =  ((C−C_0_)×D_OW_ ×SA)/(OUR×h)) and setting C, the oxygen concentration at the cell layer, to 0. The diffusivity of oxygen in cell culture medium was assumed to be the same as the diffusivity of oxygen in water, D_OW_ for the purposes of this calculation. D_OW_ at 37 degrees Celsius is 3.55×10^−5^ cm^2^s^−1^ [39]. The surface area, SA, of a well of a Nunc 6-well plate is 9.6 cm^2^ and the height of liquid was 0.21 cm. The concentration of oxygen at the surface, C_0_, is equal to the solubility multiplied by the partial pressure. The solubility of oxygen in water at 37 degrees Celsius is 0.00021 mmol/cm^3^ [40].

The maximum number of cells that can be supported before oxygen becomes limiting is 6.6×10^4^–6.71×10^4^ and 61.4×10^4^–62.4×10^4^ cells at 2% and 20% oxygen, respectively. This means that oxygen becomes limiting at the start and during the exponential growth phase at 2% oxygen and 20% oxygen, respectively, in this experimental set-up.

### Inhibition of mitochondrial complexes I and II

**Figure 5:**
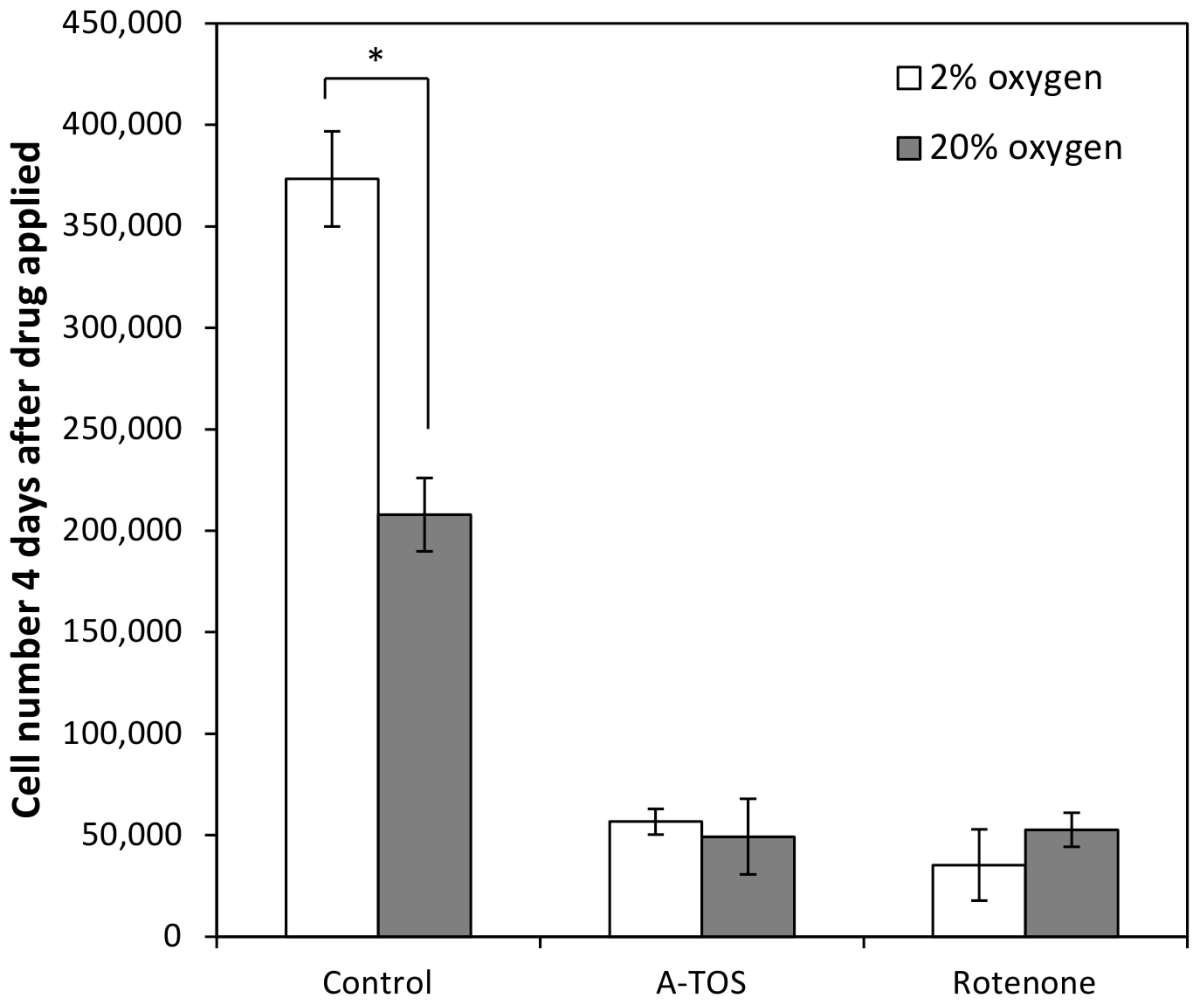
Effect of blocking complex I and II on human embryonic stem cell growth. Effect of blocking complex I and complex II on hESC growth at 20% and 2% oxygen. Plot shows the cell number per well 4 days after the addition of rotenone or D-α-tocopherol succinate (A-TOS) or to the cell culture medium block complex I and II respectively. Control has no drug added. Values are averages ± standard deviation, n = 3. * indicates a p-value <0.5, deemed statistically significant.

Mitochondrial complex I and II were blocked with rotenone to block mitochondrial complex I and α-tocopherol succinate (α-TOS) to block mitochondrial respiratory complex II to get an indication of the importance of the respiratory chain in hESCs. Blocking the complexes caused an arrest in cell growth (Figure 5). From this result, it can be inferred that the respiratory chain is essential to MEL-2 hESC expansion.

### Hypoxia inducible factor, HIF-1α

**Figure 6:**
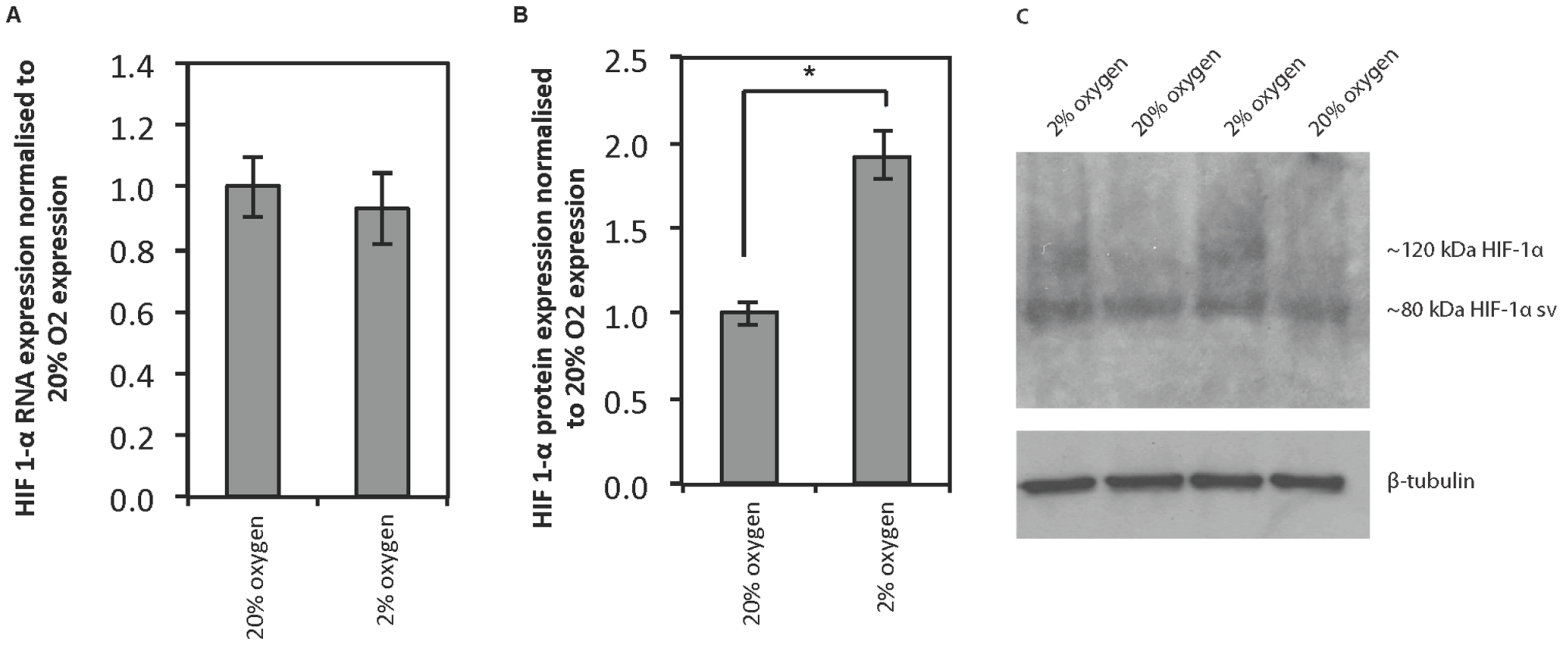
HIF-1α expression of human embryonic stem cells cultured at physiological and atmospheric oxygen concentrations. HIF-1α expression during the exponential phase of growth for hESCs cultured at 20% and 2% oxygen. A) HIF-1α RNA expression normalised to RNA expression at 20% oxygen. Values are averages ± standard deviation, n = 3. No significant difference was found between HIF-1α expression at the RNA level at 20% oxygen compared with 2% oxygen. B) Total HIF-1α protein expression normalised to protein expression at 20% oxygen. Protein expression measured by densitometry analysis of a western blot. Values are averages ± standard deviation, n = 2. * indicates a p-value <0.5, deemed statistically significant. C) Western blot showing protein probed for HIF-1α (both 120 kDa form and the 80 kDa splice variant (sv)) and β-tubulin as a loading control.

HIF-1α expression during the exponential growth phase (at the protein level) was measured by western blot and quantified by densitometry analysis. As expected HIF-1α was found to be expressed at a statistically higher level at 2% oxygen compared with 20% oxygen (Figure 6). No statistical difference was found in HIF-1α expression at the RNA level under the same conditions, in keeping with the notion that the expression of this protein is controlled by protein stability and not mRNA expression.

## Discussion

Human embryonic stem cells are considered to be highly metabolically active with a highly glycolytic nature [14]–[16]. They are also known to have fewer mitochondria than terminally differentiated cells [18] and possess mitochondria that appear immature and lack normal cristae [16], [19]. Together this has led to the preposition that the mitochondria are more inactive in hESCs than in differentiated cells and that energy generation by OXPHOS in the mitochondria is also reduced in hESCs [16]. The metabolic flux analysis conducted in this study reaffirms the highly glycolytic nature of hESCs but also shows an active citric acid cycle within the mitochondria and opportunistic use of energy substrates to maximise ATP production.

At first glance MEL-2 hESCs appear to be more metabolically active at physiological oxygen concentrations with 70% of fluxes showing significant differences between 2% and 20% oxygen being greater at 2% oxygen. In particular, large differences were noted for those fluxes associated with glycolysis, with glucose uptake being 1.8 times greater at physiological oxygen. Glycolysis is completely disconnected from the TCA cycle at both oxygen concentrations, as indicated by lactate to glucose ratios of 2.3, of a theoretical maximum of 2. The results of the MFA further support this by indicating that all of the pyruvate is converted to lactate, rather than entering the mitochondria. This phenomenon is often seen in highly proliferative cells, such as cancer cells [41]–[43] and during embryogenesis [41].

Hypotheses put forward in the literature to explain the disconnection of glycolysis from the TCA cycle in highly proliferative cells range from the phenomena being a more rapid way to produce ATP despite being less efficient [19] to it being a protection mechanism from the production of reactive oxygen species (ROS) during ATP production by OXPHOS. What is often not investigated within these studies, however, is the activity of the mitochondria and OXPHOS. Our metabolic flux analysis uniquely allowed us to reveal that mitochondrial oxidative phosphorylation in fact significantly contributes to cellular ATP production by utilising glutamine as the main carbon source. In confirmation of this result, when complex I and II, necessary for OXPHOS, were blocked there was a reduction in cell proliferation, indicating that OXPHOS is contributing to the energy demand required for the rapid proliferation of MEL-2 hESCs observed in this study. Interestingly in other cell types glutamine oxidation is increased by the mitochondrial uncoupling protein UCP2 [44], [45]. UCP2 has been shown to be repressed during hESC differentiation [46] lending further support to the idea that UCP expression may play a central role in shaping the metabolic make-up of hESC and establishing this aerobic glycolysis state.

The results of the MFA showed no statistical difference in the total ATP produced by MEL-2 hESCs but rather a difference in the flux through the internal pathways contributing to the total ATP production at 2% and 20% oxygen. The results show a greater flux through glycolysis pathways at physiological oxygen and a greater flux through OXPHOS at atmospheric oxygen concentrations. This is supported by the greater uptake of glucose at 2% oxygen and a greater uptake of glutamine at 20% oxygen. The question then is what is the molecular mechanism governing the use of different internal metabolic pathways? While answering this question will require additional studies, it is possible to suggest some potential hypotheses based on the results of this study.

In general, metabolic homeostasis is controlled by the supply of metabolites or cellular demand. All of the metabolites measured were found to be in excess throughout the culture, including glucose and glutamine and so the uptake of either energy substrate is not limited by the supply to the cell. Oxygen however was found to be limiting in cultures under both physiological and atmospheric oxygen concentrations, but becomes limiting earlier at physiological oxygen. It is then reasonable to hypothesise that oxygen supply controls the flux through OXPHOS, and that to compensate and meet the total cellular energy demand, hESCs may then increase the flux through glycolysis pathways. A requirement of an increase in flux through glycolysis would be an increase in glucose uptake. Glucose is transported into the cell by facilitated transport via the GLUT family of proteins. One of the key regulators of GLUT expression, and in fact of many proteins required for glycolysis, is HIF-1α [47]. Indeed, greater levels of HIF1-α protein were found in hESCs cultured under physiological oxygen compared with atmospheric oxygen. HIF-1α is in turn regulated by oxygen concentration within the cell and is up regulated under hypoxic conditions.

While major differences were found in the flux through energy pathways, no statistical difference was found between the growth rates of hESCs at physiological and atmospheric oxygen concentrations. Previously this point has been contested in the literature, with some groups reporting no dependence of growth rates on oxygen concentration [48] while others report that oxygen does effect cell growth rate [24]. It should be noted that in the past, however, growth rates have typically been measured indirectly by measuring expansion in colony or EB diameter. While this methodology allows for the maintenance of pluripotent cells, it does not allow for precise control of initial seeding densities. The protocols developed within this paper build on the published single cell passaging techniques and couple them with flow cytometry techniques for cell counting to allow for precise cell enumeration and accurate measurements of cellular growth rates. The similarity in growth rates observed within this study were further supported by the results of the MFA, which indicated no significant differences in the internal fluxes for reactions associated with biosynthesis at physiological and atmospheric oxygen concentrations.

In summary, the results presented in this paper, even though at present based only on a single well known hESC line, MLE-2, provide the most detailed metabolic profile of hESCs to date, providing an invaluable resource for understanding hESC metabolism. The results indicate that hESCs alter the flux through energy pathways, including OXPHOS, to maximise ATP production depending on the culture conditions. To do this hESCs utilise not only glycolysis but also OXPHOS in the mitochondria utilising glutamine as a carbon source. The results also showed no difference in the growth rates of cells cultured under physiological or atmospheric oxygen concentrations. The results may be used in the development of novel culture mediums for hESC maintenance and expansion, as well as to improve rational bioreactor design to ensure that metabolites are delivered and waste products are removed in an effective fashion. In addition, the growth profiles presented may be used to predict the fold expansion in cultures under the conditions described within this paper.

## Supporting Information

Figure S1 **Cell enumeration by flow cytometry strategy.** Cell enumeration by flow cytometry strategy. A known volume of Flow count flurospheres of a known concentration was added to a known volume of cell suspension. The sample was analysed on a BD LSR II flow cytometer. Analysis was performed using the following regions. First, the bead population was selected on the FITC 530/30 channel, shown in red. Next, the cell population was selected based on Hoechst staining on the 450/50 channel, shown in green. Finally, the multiplets were selected on a histogram of 450/50 gated to select the cell population as the population after the G2/M peak, this region is shown in blue on the dot plots.
(PDF)

Figure S2 **Karyotype analysis of human embryonic stem cell line.** Karyotype analysis was conducted on the MEL-2 hESC cell stocks at p18+2+15 after experiments were conducted. Female karyotype with no abnormalities was detected for 25 cells tested.
(PDF)

Figure S3 **Osmolality and pH of the cell culture supernatant.** The pH (A) and the osmolality (B) of the cell culture medium were measured at each time point throughout the experiment and found to be within the limits to promote normal hESC growth. Values are averages ± standard deviation, n = 6.
(PDF)

Figure S4 **Metabolite concentration profiles.** Concentration of metabolites in the cell culture media at time points throughout the experiment at physiological, 2%, and atmospheric, 20% oxygen concentrations. Values are means ± standard deviation, n = 6.
(PDF)

Table S1 **Flux through reactions considered essential for hESC metabolism.** Results of the metabolic flux analysis - The MFA model consisted of over 2000 reactions. Of these reactions 288 were considered essential for hESC metabolism. The metabolic reactions modelled within the MFA can be broken into six categories:Biosynthesis – reactions directly involved with synthesising biomass;"Amino acid catabolism;"Central pathway – metabolism pathways present in all three domains of life;"Energy – reactions involved with the production of ATP;"Transport – transport of metabolites within the cell, eg from cytoplasm to the mitochondria; and"Exchange – transport of metabolites into and out of the cell."
The flux through these reactions in hESCs cultured at both physiological and atmospheric oxygen concentrations are given in Table S1. Note: Reaction ID or component followed by ‘_mt’ indicates that the reaction takes place or the reactant is located within the mitochondria.
(PDF)

## References

[1] Oh SKW, Choo ABH (2006) Human embryonic stem cells: Technological challenges towards therapy. Clinical and Experimental Pharmacology and Physiology 33: 489–495. 16700884 10.1111/j.1440-1681.2006.04397.x

[2] Hernandez D, Ruban L, Mason C (2011) Feeder-Free Culture of Human Embryonic Stem Cells for Scalable Expansion in a Reproducible Manner. Stem Cells and Development 20: 1089–1098. 21142495 10.1089/scd.2009.0507

[3] Zweigerdt R, Olmer R, Singh H, Haverich A, Martin U (2011) Scalable expansion of human pluripotent stem cells in suspension culture. Nature Protocols 6: 689–700. 21527925 10.1038/nprot.2011.318

[4] Fong WJ, Tan HL, Choo A, Oh SKW (2005) Perfusion cultures of human embryonic stem cells. Bioprocess and Biosystems Engineering 27: 381–387. 15928928 10.1007/s00449-005-0421-5

[5] Oh SKW, Chen AK, Mok Y, Chen XL, Lim UM, et al (2009) Long-term microcarrier suspension cultures of human embryonic stem cells. Stem Cell Research 2: 219–230. 19393590 10.1016/j.scr.2009.02.005

[6] Serra M, Brito C, Sousa MFQ, Jensen J, Tostoes R, et al (2010) Improving expansion of pluripotent human embryonic stem cells in perfused bioreactors through oxygen control. Journal of Biotechnology 148: 208–215. 20600380 10.1016/j.jbiotec.2010.06.015

[7] Krawetz R, Taiani JT, Liu SY, Meng GL, Li XY, et al (2010) Large-Scale Expansion of Pluripotent Human Embryonic Stem Cells in Stirred-Suspension Bioreactors. Tissue Engineering Part C-Methods 16: 573–582. 19737071 10.1089/ten.TEC.2009.0228

[8] Ludwig TE, Levenstein ME, Jones JM, Berggren WT, Mitchen ER, et al (2006) Derivation of human embryonic stem cells in defined conditions. Nature Biotechnology 24: 185–187. 10.1038/nbt1177 16388305

[9] Wang L, Schuiz TC, Sherrer ES, Dauphin DS, Shin S, et al (2007) Self-renewal of human embryonic stem cells requires insuhn-like growth factor-1 receptor and ERBB2 receptor signaling. Blood 110: 4111–4119. 17761519 10.1182/blood-2007-03-082586 PMC2190616

[10] Lu J, Hou RH, Booth CJ, Yang SH, Snyder M (2006) Defined culture conditions of human embryonic stem cells. Proceedings of the National Academy of Sciences of the United States of America 103: 5688–5693. 16595624 10.1073/pnas.0601383103 PMC1458634

[11] Li Y, Powell S, Brunette E, Lebkowski J, Mandalam R (2005) Expansion of human embryonic stem cells in defined serum-free medium devoid of animal-derived products. Biotechnology and Bioengineering 91: 688–698. 15971228 10.1002/bit.20536

[12] Akopian V, Andrews PW, Beil S, Benvenisty N, Brehm J, et al (2010) Comparison of defined culture systems for feeder cell free propagation of human embryonic stem cells. In Vitro Cellular & Developmental Biology-Animal 46: 247–258. 20186512 10.1007/s11626-010-9297-z PMC2855804

[13] Furue MK, Na J, Jackson JP, Okamoto T, Jones M, et al (2008) Heparin promotes the growth of human embryonic stem cells in a defined serum-free medium. Proceedings of the National Academy of Sciences of the United States of America 105: 13409–13414. 18725626 10.1073/pnas.0806136105 PMC2522264

[14] Chen X, Chen A, Woo TL, Choo ABH, Reuveny S, et al. (2010) Investigations into the metabolism of two-dimensional colony and suspended microcarrier cultures of human embryonic stem cells in serum-free medium. Stem Cells and Development 19. 10.1089/scd.2010.0077 20380517

[15] Fernandes TG, Fernandes-Platzgummer AM, da Silva CL, Diogo MM, Cabral JMS (2010) Kinetic and metabolic analysis of mouse embryonic stem cell expansion under serum-free conditions. Biotechnology Letters 32: 171–179. 19705070 10.1007/s10529-009-0108-0

[16] Varum S, Rodrigues AS, Moura MB, Momclovic O, Easley CA, et al (2011) Energy metabolism in human pluripotent stem cells and their differentiated counterparts. Plos One 6: e20914–e20914. 21698063 10.1371/journal.pone.0020914 PMC3117868

[17] Zeng AP, Hu WS, Deckwer WD (1998) Variation of stoichiometric ratios and their correlation for monitoring and control of animal cell cultures. Biotechnology Progress 14: 434–441. 9622524 10.1021/bp9800337

[18] Facucho-Oliveira JM, St John JC (2009) The Relationship Between Pluripotency and Mitochondrial DNA Proliferation During Early Embryo Development and Embryonic Stem Cell Differentiation. Stem Cell Reviews and Reports 5: 140–158. 19521804 10.1007/s12015-009-9058-0

[19] Jezek P, Plecita-Hlavata L, Smolkova K, Rossignol R (2010) Distinctions and similarities of cell bioenergetics and the role of mitochondria in hypoxia, cancer, and embryonic development. International Journal of Biochemistry & Cell Biology 42: 604–622. 19931409 10.1016/j.biocel.2009.11.008

[20] Thomson JA, Itskovitz-Eldor J, Shapiro SS, Waknitz MA, Swiergiel JJ, et al (1998) Embryonic stem cell lines derived from human blastocysts. Science 282: 1145–1147. 9804556 10.1126/science.282.5391.1145

[21] Fischer B, Bavister BD (1993) Oxygen-tension in the oviduct and uterus of rhesus-monkeys, hamsters and rabbits. Journal of Reproducition and Fertility 99: 673–679. 10.1530/jrf.0.0990673 8107053

[22] Forsyth NR, Musio A, Vezzoni P, Simpson A, Noble BS, et al (2006) Physiologic oxygen enhances human embryonic stem cell clonal recovery and reduces chromosomal abnormalities. Cloning and Stem Cells 8: 16–23. 16571074 10.1089/clo.2006.8.16

[23] Wang F, Thirumangalathu S, Loeken MR (2006) Establishment of new mouse embryonic stem cell lines is improved by physiological glucose and oxygen. Cloning and Stem Cells 8: 108–116. 16776602 10.1089/clo.2006.8.108

[24] Ezashi T, Das P, Roberts RM (2005) Low O2 tensions and the prevention of differentiation of hES cells. Proceedings of the National Academy of Sciences of the United States of America 102: 4783–4788. 15772165 10.1073/pnas.0501283102 PMC554750

[25] Prasad SM, Czepiel M, Cetinkaya C, Smigielska K, Weli SC, et al (2009) Continuous hypoxic culturing maintains activation of Notch and allows long-term propagation of human embryonic stem cells without spontaneous differentiation. Cell Proliferation 42: 63–74. 19143764 10.1111/j.1365-2184.2008.00571.x PMC6496631

[26] Forristal CE, Wright KL, Hanley NA, Oreffo ROC, Houghton FD (2010) Hypoxia inducible factors regulate pluripotency and proliferation in human embryonic stem cells cultured at reduced oxygen tensions. Reproduction 139: 85–97. 19755485 10.1530/REP-09-0300 PMC2791494

[27] Adewumi O, Aflatoonian B, Ahrlund-Richter L, Amit M, Andrews PW, et al (2007) Characterization of human embryonic stem cell lines by the International Stem Cell Initiative. Nature Biotechnology 25: 803–816. 10.1038/nbt1318 17572666

[28] Costa M, Sourris K, Hatzistavrou T, Elefanty AG, Stanley EG (2008) Expansion of human embryonic stem cells in vitro. Current Protocols in Stem Cell Biology 1: 1C.1.1–1C.1.7. 10.1002/9780470151808.sc01c01s5 18770627

[29] Krömer JO, Fritz M, Heinzle E, Wittmann C (2005) In vivo quantification of intracellular amino acids and intermediates of the methionine pathway in Corynebacterium glutamicum. Analytical Biochemistry 340: 171–173. 15802143 10.1016/j.ab.2005.01.027

[30] Quek L, Dietmair S, Krömer J, Nielsen L (2010) Metabolic flux analysis in mammalian cell culture. Metabolic Engineering Journal 12: 161–171. 10.1016/j.ymben.2009.09.002 19833223

[31] Quek L, Nielsen L (2008) On the reconstruction of the Mus musculus genome-scale metabolic network model. Genome Information 21: 89–100. 19425150

[32] Bonarius HPJ, Hatzimanikatis V, Meesters KPH, deGooijer CD, Schmid G, et al (1996) Metabolic flux analysis of hybridoma cells in different culture media using mass balances. Biotechnology and Bioengineering 50: 299–318. 18626958 10.1002/(SICI)1097-0290(19960505)50:3<299::AID-BIT9>3.0.CO;2-B

[33] Dong LF, Low P, Dyason JC, Wang XF, Prochazka L, et al (2008) alpha-tocopheryl succinate induces apoptosis by targeting ubiquinone-binding sites in mitochondrial respiratory complex II. Oncogene 27: 4324–4335. 18372923 10.1038/onc.2008.69 PMC2668987

[34] Li B, Chauvin C, De Paulis D, De Oliveira F, Gharib A, et al (2012) Inhibition of complex I regulates the mitochondrial permeability transition through a phosphate-sensitive inhibitory site masked by cyclophilin D. Biochimica Et Biophysica Acta-Bioenergetics. 1817: 1628–1634. 10.1016/j.bbabio.2012.05.011 22659400

[35] Miller L (2010) Analyzing gels and western blots with ImageJ

[36] Schmittgen TD, Livak KJ (2008) Analyzing real-time PCR data by the comparative C(T) method. Nat Protoc 3: 1101–1108. 18546601 10.1038/nprot.2008.73

[37] Livak KJ, Schmittgen TD (2001) Analysis of relative gene expression data using real-time quantitative PCR and the 2(-Delta Delta C(T)) Method. Methods 25: 402–408. 11846609 10.1006/meth.2001.1262

[38] Bustin SA, Benes V, Garson JA, Hellemans J, Huggett J, et al (2009) The MIQE guidelines: minimum information for publication of quantitative real-time PCR experiments. Clin Chem 55: 611–622. 19246619 10.1373/clinchem.2008.112797

[39] (1999) Perry's chemical engineering handbook; Perry RH, Green DW, editors. New York: McGraw Hill.

[40] (2003) OxyMicro User Manual - Fibre-optic oxygen measurement systems with microsensors. In: Instruments WP, editor.

[41] Fritz V, Fajas L (2010) Metabolism and proliferation share common regulatory pathways in cancer cells. Oncogene 29: 4369–4377. 20514019 10.1038/onc.2010.182 PMC3004916

[42] Heiden MGV, Locasale JW, Swanson KD, Sharfi H, Heffron GJ, et al (2010) Evidence for an Alternative Glycolytic Pathway in Rapidly Proliferating Cells. Science 329: 1492–1499. 20847263 10.1126/science.1188015 PMC3030121

[43] Argiles JM, Lopezsoriano FJ (1990) Why do cancer-cells have such a high glycolytic rate? Medical Hypotheses 32: 151–155. 2142979 10.1016/0306-9877(90)90039-h

[44] Pecqueur C, Bui T, Gelly C, Hauchard J, Barbot C, et al (2008) Uncoupling protein-2 controls proliferation by promoting fatty acid oxidation and limiting glycolysis-derived pyruvate utilization. FASEB Journal 22: 9–18. 17855623 10.1096/fj.07-8945com

[45] Pecqueur C, Alves-Guerra C, Ricquier D, Bouillaud F (2009) UCP2, a metabolic sensor coupling glucose oxidation to mitochondrial metabolism? IUBMB Life 61: 762–767. 19514063 10.1002/iub.188

[46] Zhang J, Khvorostov I, Hong JS, Oktay Y, Vergnes L, et al (2011) UCP2 regulates energy metabolism and differentiation potential of human pluripotent stem cells. EMBO Journal 30: 4860–4873. 22085932 10.1038/emboj.2011.401 PMC3243621

[47] Airley RE, Mobasheri A (2007) Hypoxic regulation of glucose transport, anaerobic metabolism and angiogenesis in cancer: Novel pathways and targets for anticancer therapeutics. Chemotherapy 53: 233–256. 17595539 10.1159/000104457

[48] Kurosawa H, Kimura M, Noda T, Amano Y (2006) Effect of oxygen on In Vitro differentiation of mouse embryonic stem cells. Journal of Bioscience and Engineering 101: 26–30. 10.1263/jbb.101.26 16503287

