# Supplementary material for: Metabolic Profiling and Flux Analysis of MEL-2 Human Embryonic Stem Cells during Exponential Growth at Physiological and Atmospheric Oxygen Concentrations
Source: PLoS One. 2014 Nov 20;9(11):e112757. doi: 10.1371/journal.pone.0112757 (PMC4239018; doi:10.1371/journal.pone.0112757)
Supplement: Figure S1 — Cell enumeration by flow cytometry strategy. Cell enumeration by flow cytometry strategy. A known volume of Flow count flurospheres of a known concentration was added to a known volume of cell suspension. The sample was analysed on a BD LSR II flow cytometer. Analysis was performed using the following regions. First, the bead population was selected on the FITC 530/30 channel, shown in red. Next, the cell population was selected based on Hoechst staining on the 450/50 channel, shown in green. Finally, the multiplets were selected on a histogram of 450/50 gated to select the cell population as the population after the G2/M peak, this region is shown in blue on the dot plots. (PDF) [file pone.0112757.s001.pdf]

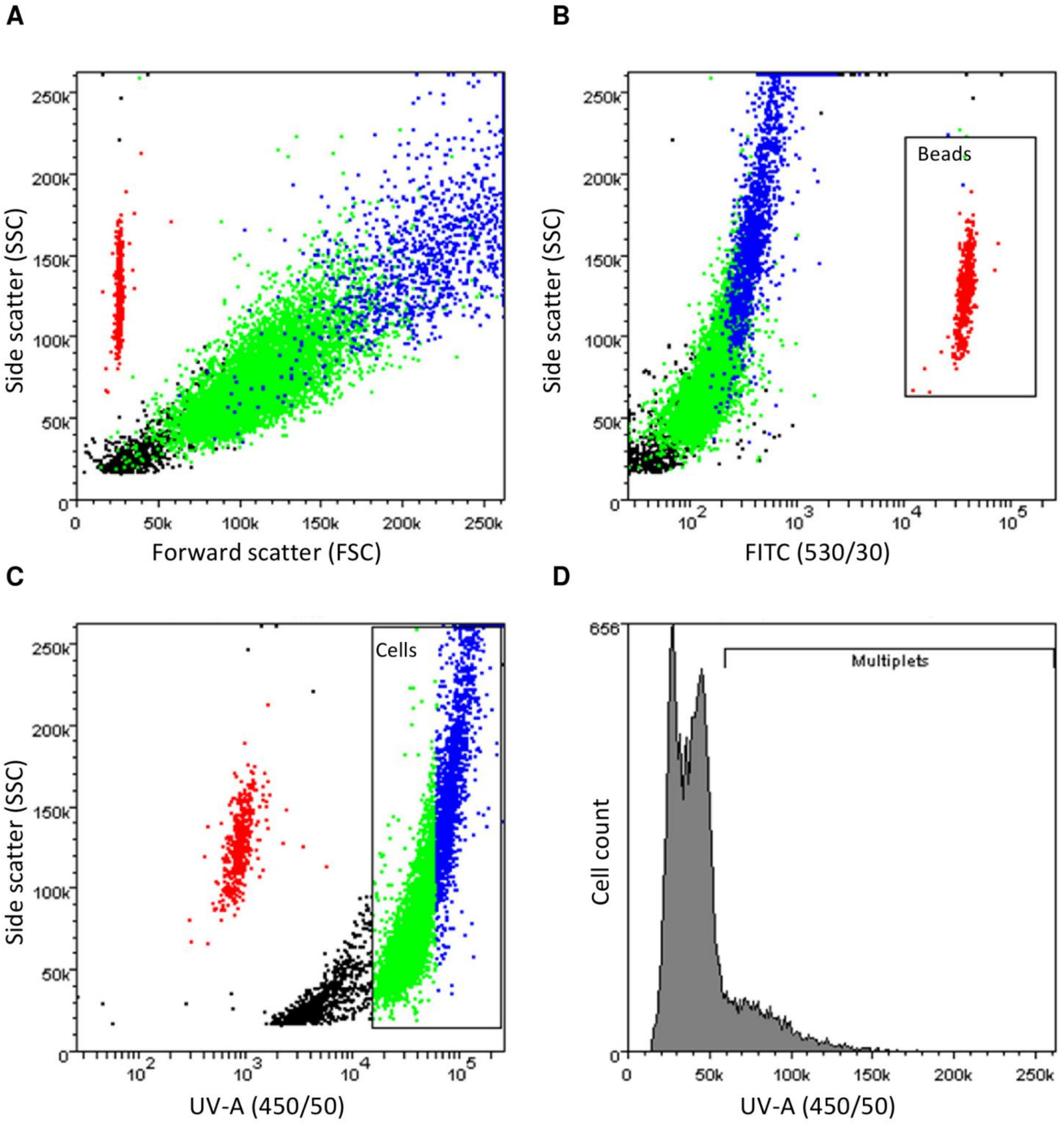

Figure S1. Cell enumeration by flow cytometry strategy.

Cell enumeration by flow cytometry strategy. A known volume of Flow count flurospheres of a known concentration was added to a known volume of cell suspension. The sample was analysed on a BD LSR II flow cytometer. Analysis was performed using the following regions. First, the bead population was selected on the FITC 530/30 channel, shown in red. Next, the cell population was selected based on Hoechst staining on the 450/50 channel, shown in green. Finally, the multiplets were selected on a histogram of 450/50 gated to select the cell population as the population after the G2/M peak, this region is shown in blue on the dot plots.
