# Supplementary material for: Metabolic Profiling and Flux Analysis of MEL-2 Human Embryonic Stem Cells during Exponential Growth at Physiological and Atmospheric Oxygen Concentrations
Source: PLoS One. 2014 Nov 20;9(11):e112757. doi: 10.1371/journal.pone.0112757 (PMC4239018; doi:10.1371/journal.pone.0112757)
Supplement: Figure S2 — Karyotype analysis of human embryonic stem cell line. Karyotype analysis was conducted on the MEL-2 hESC cell stocks at p18+2+15 after experiments were conducted. Female karyotype with no abnormalities was detected for 25 cells tested. (PDF) [file pone.0112757.s002.pdf]

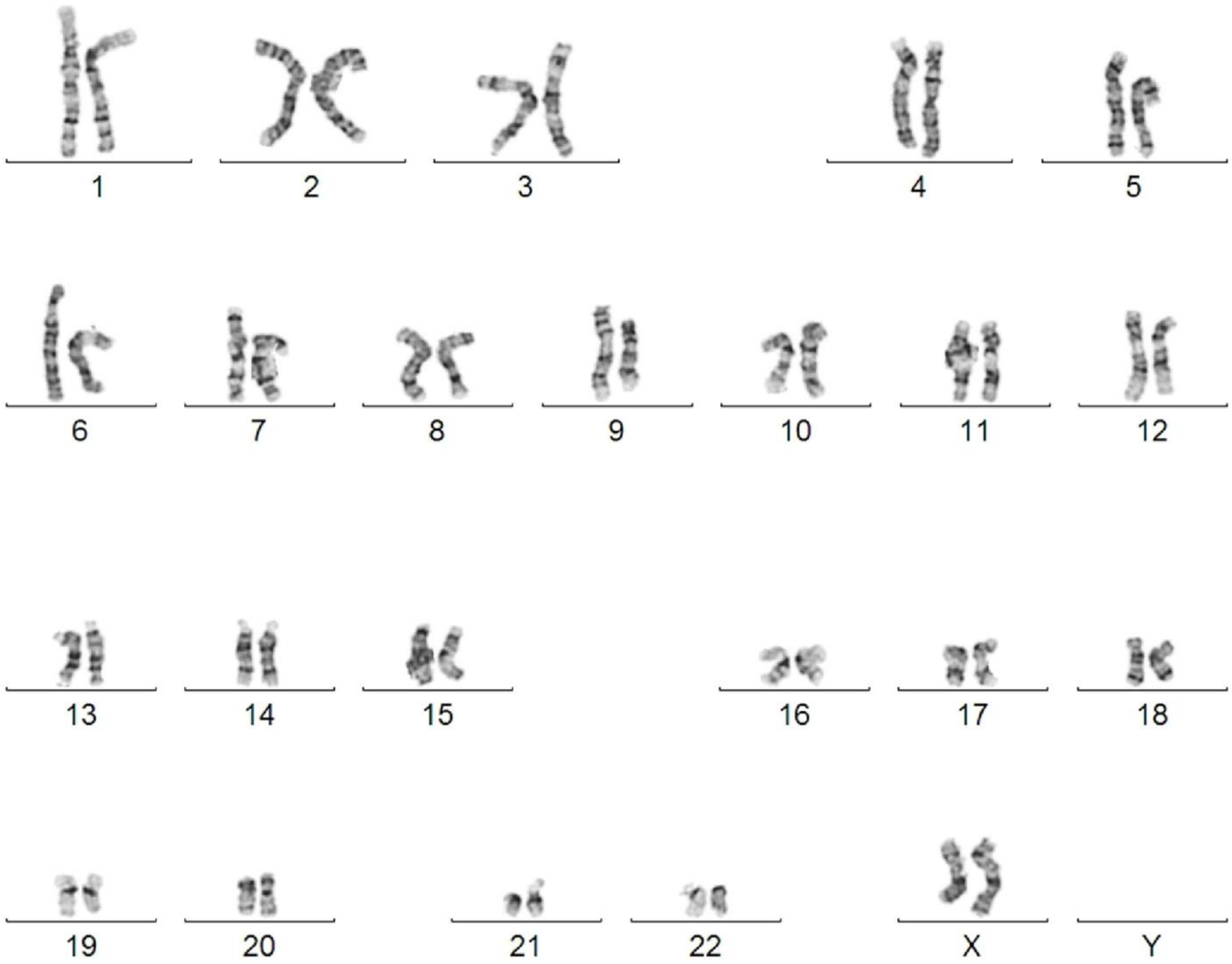

Figure S3. Karyotype analysis of human embryonic stem cell line.

Karyotype analysis was conducted on the MEL-2 hESC cell stocks at p18+2+15 after experiments were conducted. Female karyotype with no abnormalities was detected for 25 cells tested.
