# Supplementary material for: Metabolic Profiling and Flux Analysis of MEL-2 Human Embryonic Stem Cells during Exponential Growth at Physiological and Atmospheric Oxygen Concentrations
Source: PLoS One. 2014 Nov 20;9(11):e112757. doi: 10.1371/journal.pone.0112757 (PMC4239018; doi:10.1371/journal.pone.0112757)
Supplement: Figure S3 — Osmolality and pH of the cell culture supernatant. The pH (A) and the osmolality (B) of the cell culture medium were measured at each time point throughout the experiment and found to be within the limits to promote normal hESC growth. Values are averages ± standard deviation, n = 6. (PDF) [file pone.0112757.s003.pdf]

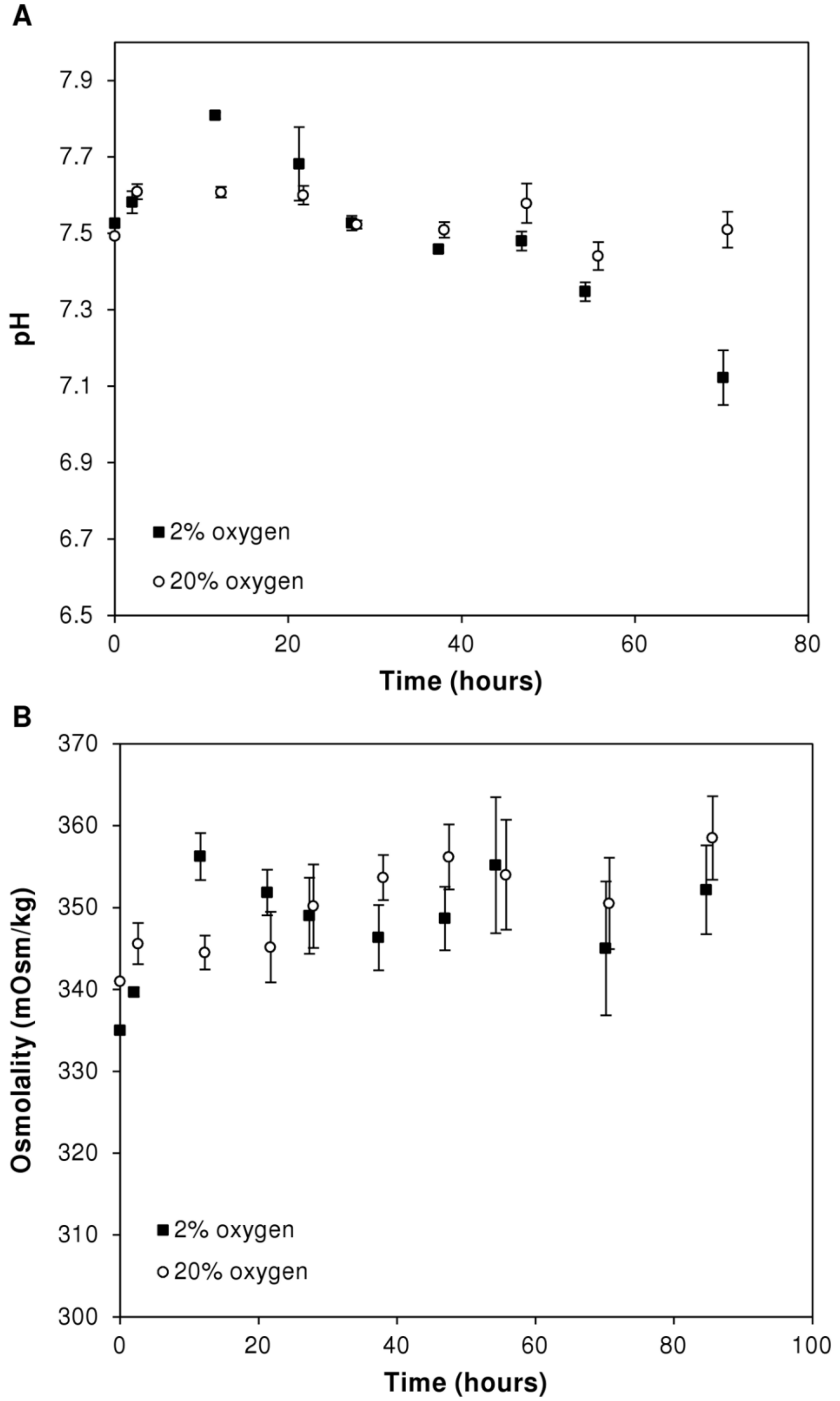

Figure S3. Osmolality and pH of the cell culture supernatant.

The pH (A) and the osmolality (B) of the cell culture medium were measured at each time point throughout the experiment and found to be within the limits to promote normal hESC growth. Values are averages ± standard deviation, n=6.
