# Supplementary material for: Metabolic Profiling and Flux Analysis of MEL-2 Human Embryonic Stem Cells during Exponential Growth at Physiological and Atmospheric Oxygen Concentrations
Source: PLoS One. 2014 Nov 20;9(11):e112757. doi: 10.1371/journal.pone.0112757 (PMC4239018; doi:10.1371/journal.pone.0112757)
Supplement: Figure S4 — Metabolite concentration profiles. Concentration of metabolites in the cell culture media at time points throughout the experiment at physiological, 2%, and atmospheric, 20% oxygen concentrations. Values are means ± standard deviation, n = 6. (PDF) [file pone.0112757.s004.pdf]

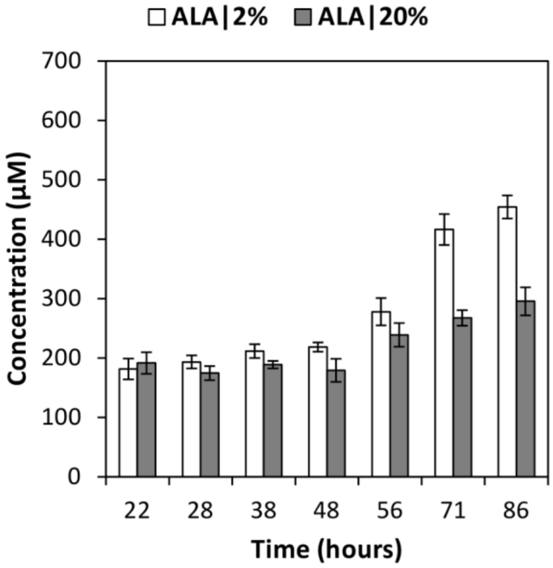
ALA|2%
ALA|20%
Concentration (µM)
Time (hours)
0 100 200 300 400 500 600 700
22 28 38 48 56 71 86

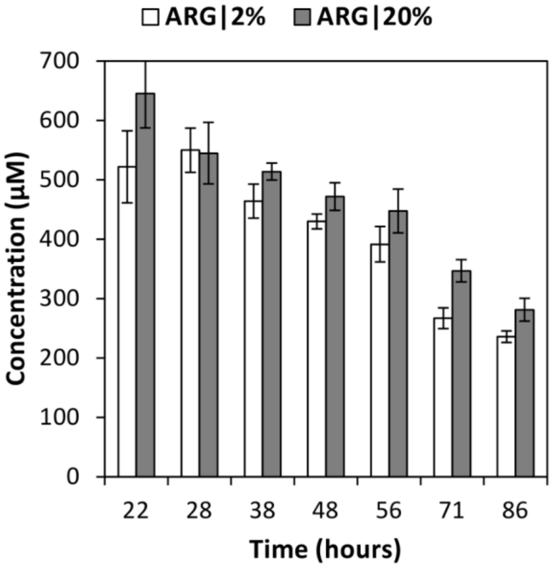
ARG|2%
ARG|20%
Concentration (µM)
Time (hours)
0 100 200 300 400 500 600 700
22 28 38 48 56 71 86

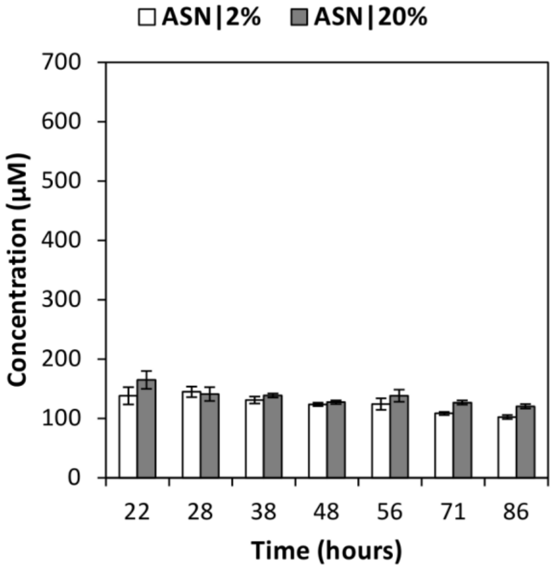
ASN|2%
ASN|20%
Concentration (µM)
Time (hours)
0 100 200 300 400 500 600 700
22 28 38 48 56 71 86

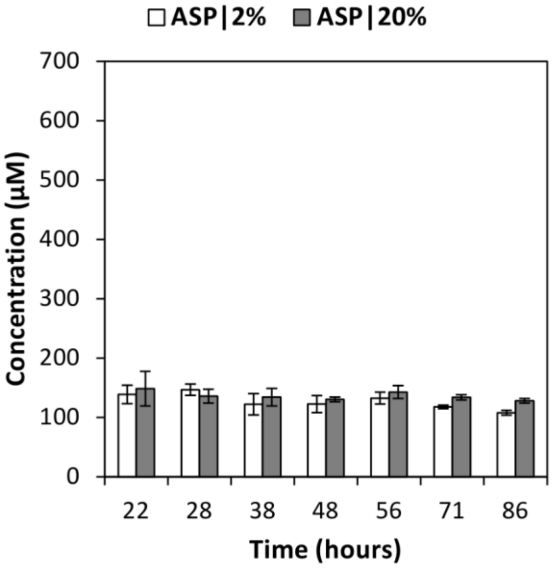
ASP|2%
ASP|20%
Concentration (µM)
Time (hours)
0 100 200 300 400 500 600 700
22 28 38 48 56 71 86

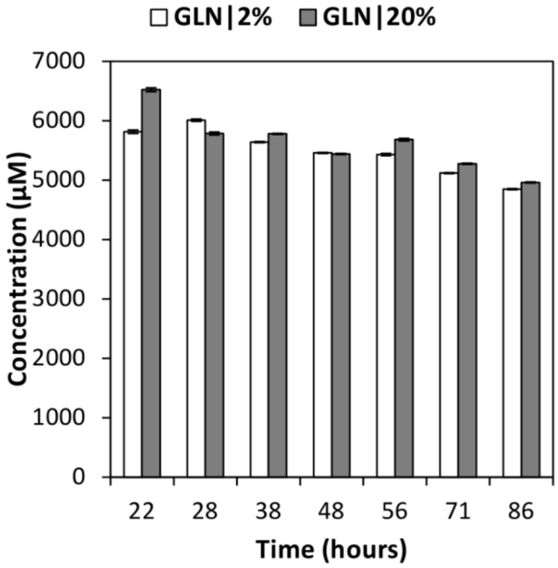
GLN|2%
GLN|20%
Concentration (µM)
Time (hours)
0 1000 2000 3000 4000 5000 6000 7000
22 28 38 48 56 71 86

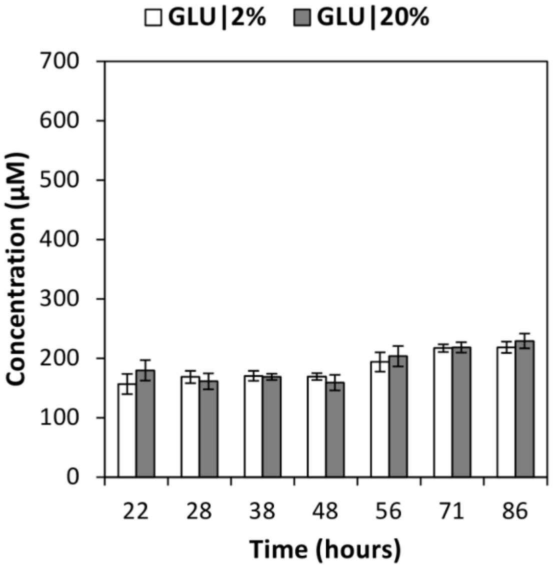
GLU|2%
GLU|20%
Concentration (µM)
Time (hours)
0 100 200 300 400 500 600 700
22 28 38 48 56 71 86

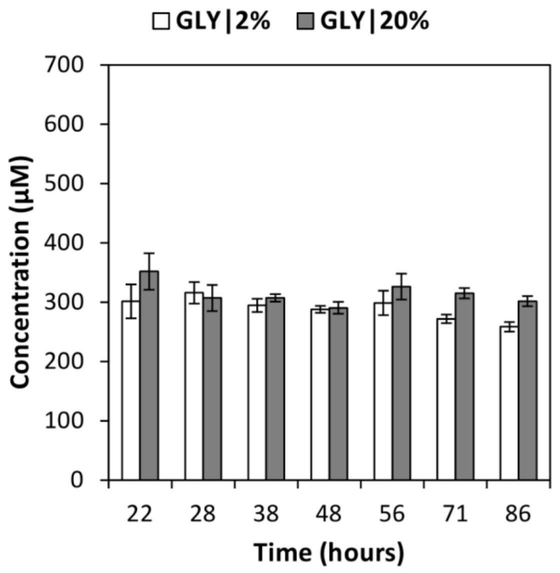
GLY|2%
GLY|20%
Concentration (µM)
0
100
200
300
400
500
600
700
22
28
38
48
56
71
86
Time (hours)

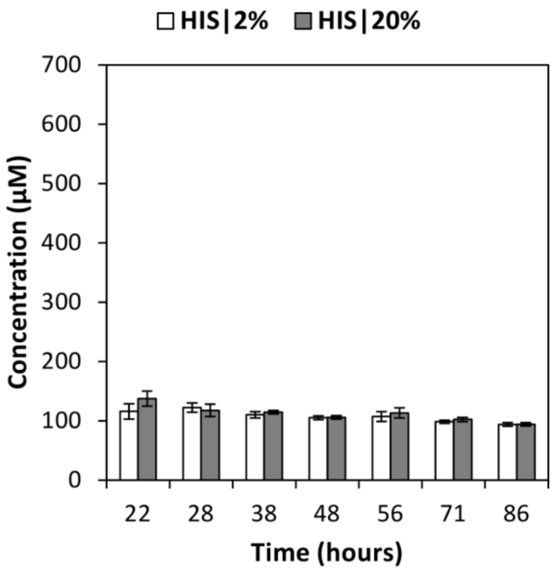
HIS|2%
HIS|20%
Concentration (µM)
0
100
200
300
400
500
600
700
22
28
38
48
56
71
86
Time (hours)

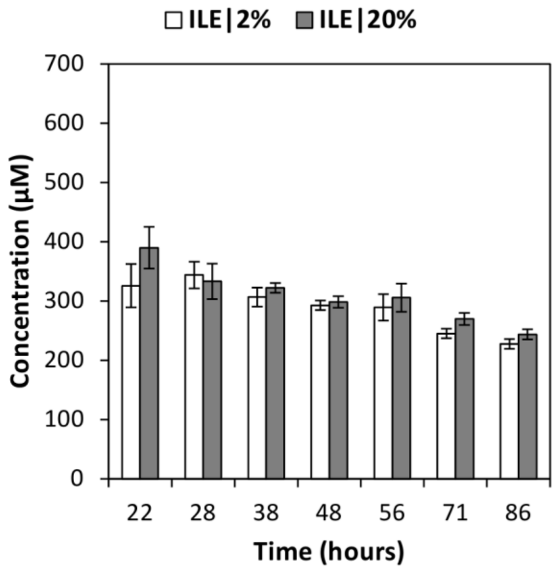
ILE|2%
ILE|20%
Concentration (µM)
0
100
200
300
400
500
600
700
22
28
38
48
56
71
86
Time (hours)

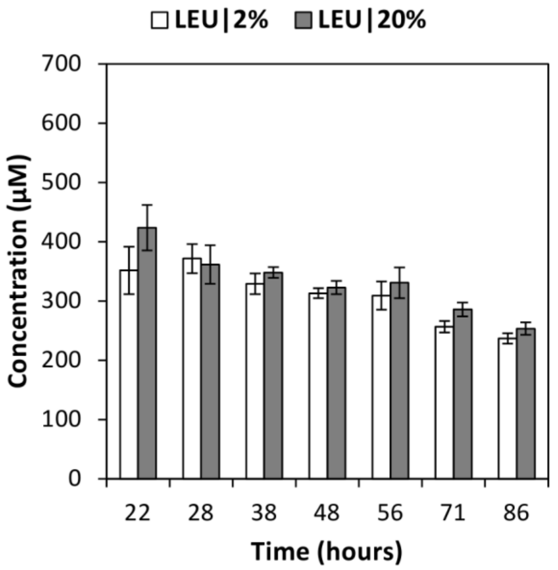
LEU|2%
LEU|20%
Concentration (µM)
0
100
200
300
400
500
600
700
22
28
38
48
56
71
86
Time (hours)

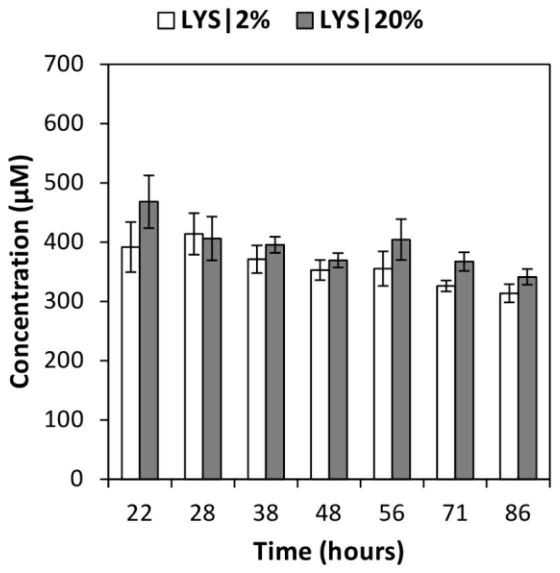
LYS|2%
LYS|20%
Concentration (µM)
0
100
200
300
400
500
600
700
22
28
38
48
56
71
86
Time (hours)

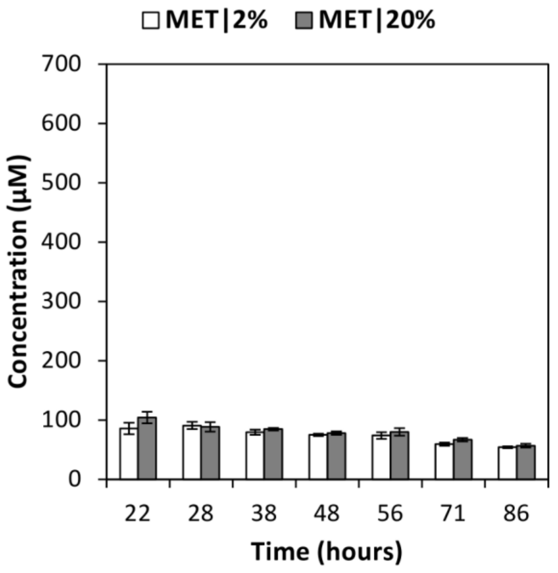
MET|2%
MET|20%
Concentration (µM)
0
100
200
300
400
500
600
700
22
28
38
48
56
71
86
Time (hours)

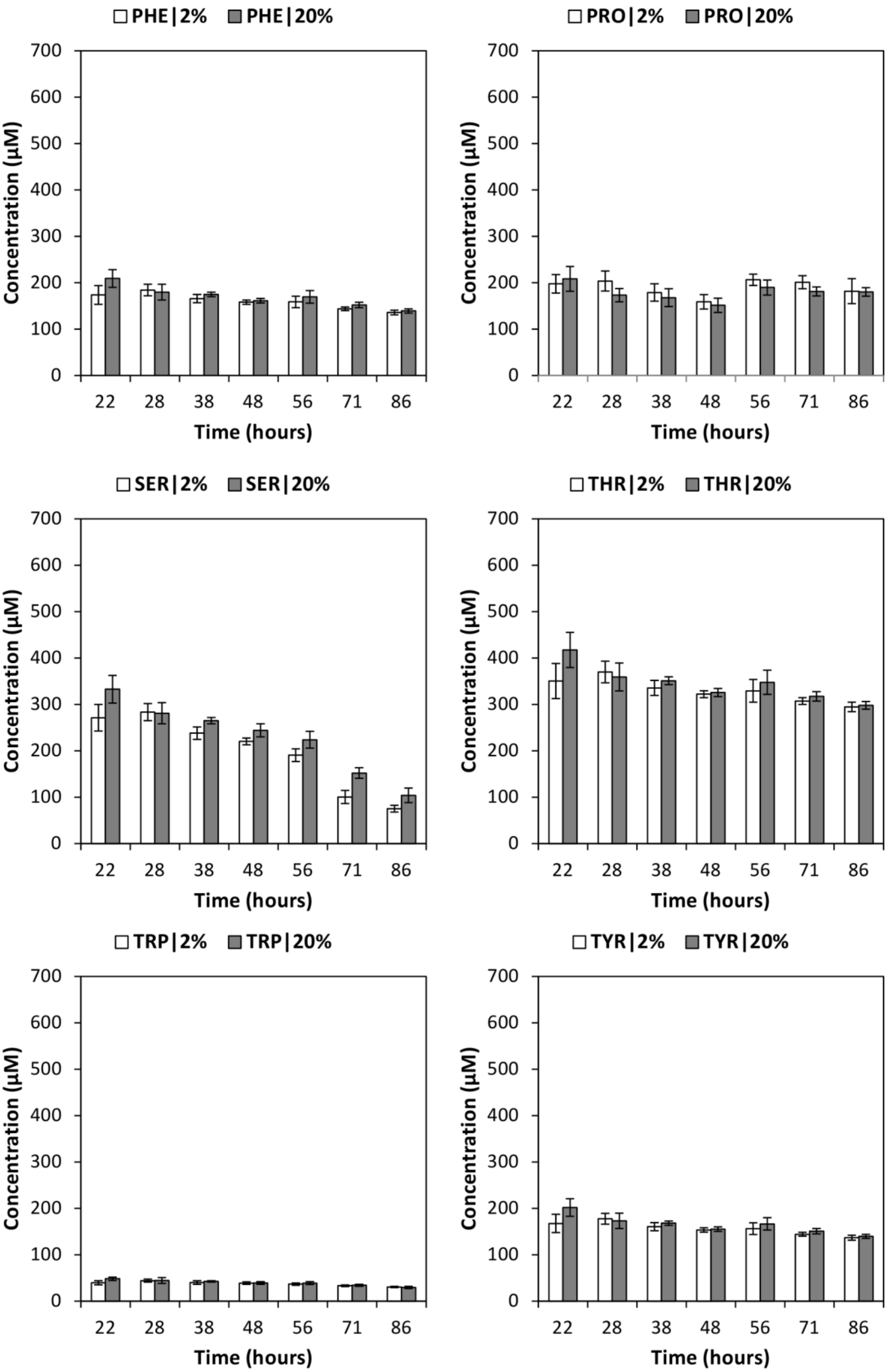
PHE|2%
PHE|20%
PRO|2%
PRO|20%
SER|2%
SER|20%
THR|2%
THR|20%
TRP|2%
TRP|20%
TYR|2%
TYR|20%
Concentration (μM)
0
100
200
300
400
500
600
700
22
28
38
48
56
71
86
Time (hours)

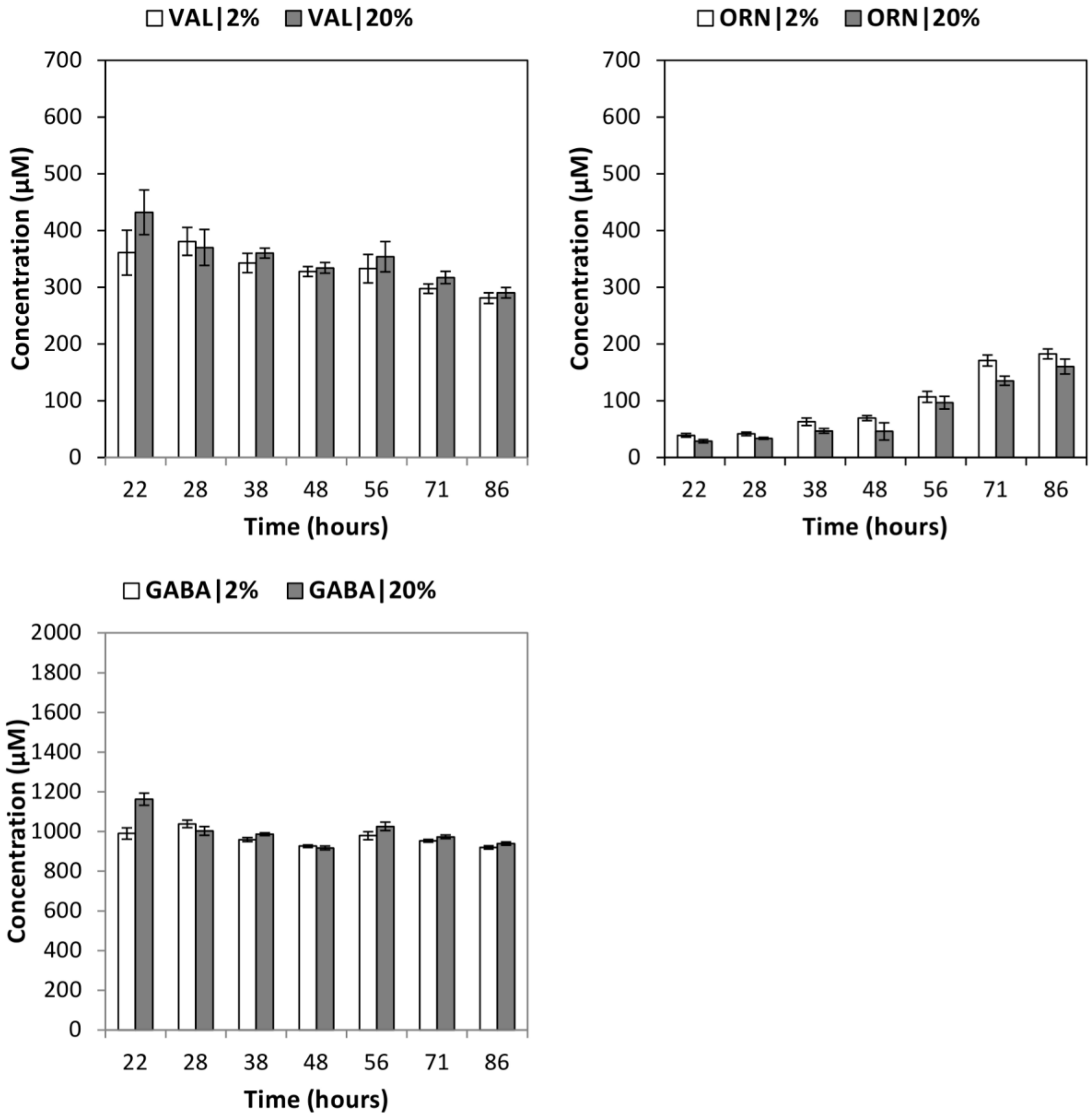

Figure S4. Metabolite concentration profiles.

Concentration of metabolites in the cell culture media at time points throughout the experiment at physiological, 2%, and atmospheric, 20% oxygen concentrations. Values are means ± standard deviation, n=6.
