# Supplementary material for: Metabolic Profiling and Flux Analysis of MEL-2 Human Embryonic Stem Cells during Exponential Growth at Physiological and Atmospheric Oxygen Concentrations
Source: PLoS One. 2014 Nov 20;9(11):e112757. doi: 10.1371/journal.pone.0112757 (PMC4239018; doi:10.1371/journal.pone.0112757)
Supplement: Table S1 — Flux through reactions considered essential for hESC metabolism. Results of the metabolic flux analysis - The MFA model consisted of over 2000 reactions. Of these reactions 288 were considered essential for hESC metabolism. The metabolic reactions modelled within the MFA can be broken into six categories:Biosynthesis – reactions directly involved with synthesising biomass;"Amino acid catabolism;"Central pathway – metabolism pathways present in all three domains of life;"Energy – reactions involved with the production of ATP;"Transport – transport of metabolites within the cell, eg from cytoplasm to the mitochondria; and"Exchange – transport of metabolites into and out of the cell." The flux through these reactions in hESCs cultured at both physiological and atmospheric oxygen concentrations are given in Table S1. Note: Reaction ID or component followed by ‘_mt’ indicates that the reaction takes place or the reactant is located within the mitochondria. (PDF) [file pone.0112757.s005.pdf]

Results of the metabolic flux analysis

The MFA model consisted of over 2000 reactions. Of these reactions 288 were considered essential for hESC metabolism. The metabolic reactions modelled within the MFA can be broken into six categories;

- Biosynthesis – reactions directly involved with synthesising biomass;
- Amino acid catabolism;
- Central pathway – metabolism pathways present in all three domains of life;
- Energy – reactions involved with the production of ATP;
- Transport – transport of metabolites within the cell, eg from cytoplasm to the mitochondria; and
- Exchange – transport of metabolites into and out of the cell.

The flux through these reactions in hESCs cultured at both physiological and atmospheric oxygen concentrations are given in Table S1.

Note: Reaction ID or component followed by '_mt' indicates that the reaction takes place or the reactant is located within the mitochondria.

Table S1. Flux through reactions considered essential for hESC metabolism

Reaction ID	Reaction	Category	Flux at 2% oxygen (mmol/gDW) Lower & upper bound		Flux at 20% oxygen (mmol/gDW) Lower & upper bound		Significant difference
EF0001	alpha-D-Glucose_ext = alpha-D-Glucose	Exchange	53.06	53.10	33.16	33.21	Yes
EF0002	ATP + H2O = ADP + Orthophosphate	Energy	215.00	216.40	218.00	219.53	Yes
EF0003	CO2 = CO2_ext	Exchange	32.73	33.15	43.68	44.14	Yes
EF0004	(S)-Lactate = (S)-Lactate_ext	Exchange	101.31	101.40	66.35	66.45	Yes
EF0005	Oxygen_ext = Oxygen	Exchange	31.23	31.75	39.85	40.46	Yes
EF0006	L-Alanine = L-Alanine_ext	Exchange	1.92	2.01	0.74	0.84	Yes
EF0007	L-Aspartate_ext = L-Aspartate	Exchange	0.17	0.26	0.01	0.10	Yes
EF0008	L-Asparagine_ext = L-Asparagine	Exchange	0.24	0.31	0.13	0.21	Yes
EF0009	L-Glutamine_ext = L-Glutamine	Exchange	3.06	3.12	8.05	8.12	Yes
EF0010	L-Glutamate = L-Glutamate_ext	Exchange	0.43	0.49	0.41	0.48	No
EF0011	L-Serine_ext = L-Serine	Exchange	1.54	1.65	1.30	1.41	Yes
EF0012	NH3 = NH3_ext	Exchange	9.49	10.12	19.25	19.89	Yes
EF0015	H2O_ext = H2O	Exchange	-37.48	-36.20	-34.23	-32.63	Yes
EF0017	L-Valine_ext = L-Valine	Exchange	0.65	0.70	0.67	0.72	No
EF0018	L-Isoleucine_ext = L-Isoleucine	Exchange	0.81	0.85	0.74	0.78	Yes
EF0019	L-Leucine_ext = L-Leucine	Exchange	0.96	1.00	0.89	0.93	Yes
EF0020	L-Arginine_ext = L-Arginine	Exchange	2.42	2.47	2.10	2.16	Yes
EF0021	L-Threonine_ext = L-Threonine	Exchange	0.44	0.51	0.52	0.59	Yes
EF0022	L-Lysine_ext = L-Lysine	Exchange	0.70	0.74	0.56	0.61	Yes
EF0023	L-Cysteine_ext = L-Cysteine	Exchange	0.16	0.27	0.14	0.26	No
EF0024	L-Methionine_ext = L-Methionine	Exchange	0.22	0.27	0.20	0.26	No
EF0025	L-Phenylalanine_ext = L-Phenylalanine	Exchange	0.30	0.34	0.31	0.36	No
EF0026	L-Tyrosine_ext = L-Tyrosine	Exchange	0.24	0.28	0.27	0.31	No
EF0027	L-Tryptophan_ext = L-Tryptophan	Exchange	0.07	0.09	0.09	0.11	Yes
EF0028	L-Histidine_ext = L-Histidine	Exchange	0.14	0.20	0.14	0.21	No
EF0029	Urea = Urea_ext	Exchange	2.15	2.52	1.84	2.22	No
BIO003	1-Phosphatidyl-D-myo-inositol = 1-Phosphatidyl-D-myo-inositol_biomass	Biosynthesis	0.02	0.02	0.02	0.02	No
BIO004	Phosphatidylcholine = Phosphatidylcholine_biomass	Biosynthesis	0.13	0.13	0.13	0.13	No
BIO005	Phosphatidylserine = Phosphatidylserine_biomass	Biosynthesis	0.01	0.01	0.01	0.01	No
BIO006	Phosphatidylethanolamine = Phosphatidylethanolamine_biomass	Biosynthesis	0.05	0.05	0.05	0.05	No
BIO007	Cardiolipin = Cardiolipin_biomass	Biosynthesis	0.01	0.01	0.01	0.01	No
BIO008	ATP = ATP_biomass + Pyrophosphate	Biosynthesis	0.06	0.06	0.06	0.06	No
BIO009	GTP = GTP_biomass + Pyrophosphate	Biosynthesis	0.11	0.11	0.11	0.11	No
BIO010	dATP = dATP_biomass + Pyrophosphate	Biosynthesis	0.03	0.03	0.03	0.03	No
BIO011	dGTP = dGTP_biomass + Pyrophosphate	Biosynthesis	0.02	0.02	0.02	0.02	No
BIO012	CTP = CTP_biomass + Pyrophosphate	Biosynthesis	0.10	0.10	0.10	0.10	No
BIO013	UTP = UTP_biomass + Pyrophosphate	Biosynthesis	0.06	0.06	0.06	0.06	No
BIO014	dCTP = dCTP_biomass + Pyrophosphate	Biosynthesis	0.02	0.02	0.02	0.02	No

Reaction ID	Reaction	Category	Flux at 2% oxygen (mmol/gDW) Lower & upper bound		Flux at 20% oxygen (mmol/gDW) Lower & upper bound		Significant difference
BIO015	dTTP = dTTP_biomass + Pyrophosphate	Biosynthesis	0.03	0.03	0.03	0.03	No
BIO016	Cholesterol = Cholesterol_biomass	Biosynthesis	0.03	0.03	0.03	0.03	No
BIO017	Sphingomyelin = Sphingomyelin_biomass	Biosynthesis	0.02	0.02	0.02	0.02	No
BIO018	Phosphatidylglycerol = Phosphatidylglycerol_biomass	Biosynthesis	0.00	0.00	0.00	0.00	Yes
BIO019	Amylose = Amylose_biomass	Biosynthesis	0.34	0.34	0.34	0.34	No
BIO028	0.468 L-Alanyl-tRNA + 0.28 L-Aspartyl-tRNA(Asn) + 0.225 L-Asparaginyl-tRNA(Asn) + 0.301 L-Glutamyl-tRNA(Gln) + 0.251 Glutaminyl-tRNA + 0.42 Glycyl-tRNA(Gly) + 0.244 L-Prolyl-tRNA(Pro) + 0.336 L-Seryl-tRNA(Ser) + 0.325 L-Valyl-tRNA(Val) + 0.253 L-Isoleucyl-tRNA(Ile) + 0.44 L-Leucyl-tRNA + 0.294 L-Arginyl-tRNA(Arg) + 0.301 L-Threonyl-tRNA(Thr) + 0.445 L-Lysyl-tRNA + 0.113 L-Cysteinyl-tRNA(Cys) + 0.108 L-Methionyl-tRNA + 0.171 L-Phenylalanyl-tRNA(Phe) + 0.142 L-Tyrosyl-tRNA(Tyr) + 0.034 L-Tryptophanyl-tRNA(Trp) + 0.112 L-Histidyl-tRNA(His) + 0.021 1-Phosphatidyl-D-myo-inositol_biomass + 0.144 Phosphatidylcholine_biomass + 0.006 Phosphatidylserine_biomass + 0.054 Phosphatidylethanolamine_biomass + 0.006 Cardiolipin_biomass + 0.064 ATP_biomass + 0.121 GTP_biomass + 0.037 dATP_biomass + 0.025 dGTP_biomass + 0.107 CTP_biomass + 0.064 UTP_biomass + 0.025 dCTP_biomass + 0.037 dTTP_biomass + 0.038 Cholesterol_biomass + 0.017 Sphingomyelin_biomass + 0.002 Phosphatidylglycerol_biomass + 0.374 Amylose_biomass + 12.14 ATP = Biomass + 12.14 ADP + 12.14 Orthophosphate	Biosynthesis	0.90	0.90	0.90	0.91	No
R02918	ATP + L-Tyrosine + tRNA(Tyr) = AMP + Pyrophosphate + L-Tyrosyl-tRNA(Tyr)	Biosynthesis	0.13	0.13	0.13	0.13	No
R03038	ATP + L-Alanine + tRNA(Ala) = AMP + Pyrophosphate + L-Alanyl-tRNA	Biosynthesis	0.42	0.42	0.42	0.42	No
R03646	ATP + L-Arginine + tRNA(Arg) = AMP + Pyrophosphate + L-Arginyl-tRNA(Arg)	Biosynthesis	0.26	0.27	0.26	0.27	No
R03647	ATP + L-Aspartate + tRNA(Asn) = AMP + Pyrophosphate + L-Aspartyl-tRNA(Asn)	Biosynthesis	0.25	0.25	0.25	0.25	No
R03648	ATP + L-Asparagine + tRNA(Asn) = AMP + Pyrophosphate + L-Asparaginyl-tRNA(Asn)	Biosynthesis	0.20	0.20	0.20	0.20	No
R03650	ATP + L-Cysteine + tRNA(Cys) = AMP + Pyrophosphate + L-Cysteinyl-tRNA(Cys)	Biosynthesis	0.10	0.10	0.10	0.10	No
R03651	ATP + L-Glutamate + tRNA(Gln) = AMP + Pyrophosphate + L-Glutamyl-tRNA(Gln)	Biosynthesis	0.27	0.27	0.27	0.27	No
R03652	ATP + L-Glutamine + tRNA(Gln) = AMP + Pyrophosphate + Glutaminyl-tRNA	Biosynthesis	0.23	0.23	0.23	0.23	No
R03654	ATP + Glycine + tRNA(Gly) = AMP + Pyrophosphate + Glycyl-tRNA(Gly)	Biosynthesis	0.38	0.38	0.38	0.38	No
R03655	ATP + L-Histidine + tRNA(His) = AMP + Pyrophosphate + L-Histidyl-tRNA(His)	Biosynthesis	0.10	0.10	0.10	0.10	No
R03656	ATP + L-Isoleucine + tRNA(Ile) = AMP + Pyrophosphate + L-Isoleucyl-tRNA(Ile)	Biosynthesis	0.23	0.23	0.23	0.23	No
R03657	ATP + L-Leucine + tRNA(Leu) = AMP + Pyrophosphate + L-Leucyl-tRNA	Biosynthesis	0.40	0.40	0.40	0.40	No
R03658	ATP + L-Lysine + tRNA(Lys) = AMP + Pyrophosphate + L-Lysyl-tRNA	Biosynthesis	0.40	0.40	0.40	0.40	No
R03659	ATP + L-Methionine + tRNA(Met) = AMP + Pyrophosphate + L-Methionyl-tRNA	Biosynthesis	0.10	0.10	0.10	0.10	No
R03660	ATP + L-Phenylalanine + tRNA(Phe) = AMP + Pyrophosphate + L-Phenylalanyl-tRNA(Phe)	Biosynthesis	0.15	0.15	0.15	0.15	No
R03661	ATP + L-Proline + tRNA(Pro) = AMP + Pyrophosphate + L-Prolyl-tRNA(Pro)	Biosynthesis	0.22	0.22	0.22	0.22	No
R03662	ATP + L-Serine + tRNA(Ser) = AMP + Pyrophosphate + L-Seryl-tRNA(Ser)	Biosynthesis	0.30	0.30	0.30	0.30	No
R03663	ATP + L-Threonine + tRNA(Thr) = AMP + Pyrophosphate + L-Threonyl-tRNA(Thr)	Biosynthesis	0.27	0.27	0.27	0.27	No
R03664	ATP + L-Tryptophan + tRNA(Trp) = AMP + Pyrophosphate + L-Tryptophanyl-tRNA(Trp)	Biosynthesis	0.03	0.03	0.03	0.03	No
R03665	ATP + L-Valine + tRNA(Val) = AMP + Pyrophosphate + L-Valyl-tRNA(Val)	Biosynthesis	0.29	0.29	0.29	0.29	No
TF0004	L-Glutamine = L-Glutamine_mt	Transport	1.27	1.99	6.21	6.99	Yes
TF0008	Oxygen = Oxygen_mt	Transport	23.80	29.78	29.32	38.25	No

Reaction ID	Reaction	Category	Flux at 2% oxygen (mmol/gDW) Lower & upper bound		Flux at 20% oxygen (mmol/gDW) Lower & upper bound		Significant difference
TF0009	CO2 = CO2_mt	Transport	-28.06	-10.97	-38.81	-12.89	No
TF0010	ATP + ADP_mt = ATP_mt + ADP	Transport	-149.75	-115.69	-192.88	-141.21	No
TF0013	H2O = H2O_mt	Transport	-173.24	-141.65	-211.50	-169.94	No
TF0014	NH3 = NH3_mt	Transport	-9.05	-7.00	-19.03	-17.32	Yes
TF0017	L-Valine = L-Valine_mt	Transport	0.36	0.41	0.37	0.43	No
TF0018	L-Leucine = L-Leucine_mt	Transport	0.56	0.60	0.49	0.53	Yes
TF0019	L-Isoleucine = L-Isoleucine_mt	Transport	0.58	0.62	0.51	0.55	Yes
TF0022	L-Ornithine = L-Ornithine_mt	Transport	1.79	2.21	1.46	1.90	No
TF0024	L-Threonine = L-Threonine_mt	Transport	0.17	0.24	0.24	0.32	Yes
TF0025	L-Lysine = L-Lysine_mt	Transport	0.30	0.34	0.16	0.21	Yes
TF0026	L-2-Aminoadipate 6-semialdehyde = L-2-Aminoadipate 6-semialdehyde_mt	Transport	-0.34	-0.30	-0.21	-0.16	Yes
TF0028	Acetoacetate = Acetoacetate_mt	Transport	-5.59	-0.94	-6.04	-0.93	No
TF0029	2-Oxobutanoate = 2-Oxobutanoate_mt	Transport	0.12	0.17	0.10	0.16	No
gCat03	Hydantoin-5-propionate + Oxygen = N-Carbamyl-L-glutamate	AA catabolism	0.04	0.10	0.04	0.11	No
gCat04	N-Carbamyl-L-glutamate = L-Glutamate + NH3 + CO2	AA catabolism	0.04	0.10	0.04	0.11	No
gCat07_mt	CO2_mt + H2O_mt = HCO3-_mt + H+_mt	Central pathway	1.75	3.48	1.62	3.21	No
gCat07	CO2 + H2O = HCO3- + H+	Central pathway	3.88	5.56	3.88	5.42	No
K00006_mt	NADH_mt + 0.5 Oxygen_mt + 2.5 ADP_mt + 2.5 Orthophosphate_mt + H+_mt = 2.5 ATP_mt + 3.5 H2O_mt + NAD+_mt	Energy	44.77	47.56	55.99	59.05	Yes
K00007_mt	FADH2_mt + 0.5 Oxygen_mt + 1.5 ADP_mt + 1.5 Orthophosphate_mt = 1.5 ATP_mt + 2.5 H2O_mt + FAD_mt	Energy	2.74	14.65	2.55	20.38	No
K00008	(9Z)-Octadecenoic acid = Fatty acid	Biosynthesis	0.45	0.45	0.45	0.45	No
K00009	ATP + Fatty acid + CoA = AMP + Acyl-CoA + Pyrophosphate	Biosynthesis	0.45	1.14	0.45	1.21	No
K00010	Acceptor + FADH2 = Reduced acceptor + FAD	Energy	0.06	0.54	0.17	0.55	No
R00004	Pyrophosphate + H2O = 2 Orthophosphate	Central pathway	7.40	8.31	7.40	8.38	No
R00069	Oxygen + 2 4-Imidazolone-5-propanoate = 2 Hydantoin-5-propionate	AA catabolism	0.02	0.05	0.02	0.05	No
R00156	ATP + UDP = ADP + UTP	Biosynthesis	0.51	8.20	0.51	8.33	No
R00158	ATP + UMP = ADP + UDP	Biosynthesis	0.18	1.61	0.18	1.74	No
R00177	ATP + L-Methionine + H2O = Orthophosphate + Pyrophosphate + S-Adenosyl-L-methionine	Central pathway	0.43	0.56	0.43	0.57	No
R00192	S-Adenosyl-L-homocysteine + H2O = Adenosine + L-Homocysteine	AA catabolism	0.43	0.56	0.44	0.57	No
R00200	ADP + Phosphoenolpyruvate = ATP + Pyruvate	Central pathway	103.29	109.82	63.58	74.17	Yes
R00238_mt	2 Acetyl-CoA_mt = CoA_mt + Acetoacetyl-CoA_mt	Biosynthesis	0.33	4.59	0.40	5.21	No
R00238	2 Acetyl-CoA = CoA + Acetoacetyl-CoA	Biosynthesis	-5.73	-1.47	-6.22	-1.40	No
R00243_mt	L-Glutamate_mt + NAD+_mt + H2O_mt = 2-Oxoglutarate_mt + NH3_mt + NADH_mt + H+_mt	AA catabolism	5.01	6.94	10.33	11.89	Yes
R00256_mt	L-Glutamine_mt + H2O_mt = L-Glutamate_mt + NH3_mt	AA catabolism	1.27	1.99	6.21	6.99	Yes
R00289	UTP + alpha-D-Glucose 1-phosphate = Pyrophosphate + UDP-glucose	Biosynthesis	0.34	1.03	0.34	1.10	No
R00292	UDP-glucose = UDP + Amylose	Biosynthesis	0.34	0.34	0.34	0.34	No
R00332	ATP + GMP = ADP + GDP	Biosynthesis	0.11	1.53	0.11	1.67	No
R00342_mt	(S)-Malate_mt + NAD+_mt = Oxaloacetate_mt + NADH_mt + H+_mt	Central pathway	7.40	27.10	3.62	31.30	No
R00410	Succinyl-CoA + Acetoacetate = Succinate + Acetoacetyl-CoA	AA catabolism	1.28	5.93	1.32	6.43	No

Reaction ID	Reaction	Category	Flux at 2% oxygen (mmol/gDW) Lower & upper bound		Flux at 20% oxygen (mmol/gDW) Lower & upper bound		Significant difference
R00412_mt	FAD_mt + Succinate_mt = FADH2_mt + Fumarate_mt	Central pathway	1.15	12.20	1.08	17.74	No
R00551	L-Arginine + H2O = L-Ornithine + Urea	AA catabolism	2.15	2.21	1.84	1.90	Yes
R00573	ATP + UTP + L-Glutamine + H2O = ADP + Orthophosphate + CTP + L-Glutamate	Biosynthesis	0.12	0.83	0.12	0.90	No
R00575	2 ATP + L-Glutamine + HCO3- + H2O = 2 ADP + Orthophosphate + L-Glutamate + Carbamoyl phosphate	Biosynthesis	0.21	0.21	0.21	0.21	No
R00658	2-Phospho-D-glycerate = Phosphoenolpyruvate + H2O	Central pathway	103.29	105.01	63.58	65.18	Yes
R00667_mt	L-Ornithine_mt + 2-Oxoglutarate_mt = L-Glutamate 5-semialdehyde_mt + L-Glutamate_mt	AA catabolism	1.79	2.21	1.46	1.90	No
R00678	L-Tryptophan + Oxygen = L-Formylkynurenine	AA catabolism	0.04	0.06	0.06	0.08	No
R00702	2 trans,trans-Farnesyl diphosphate = Pyrophosphate + Presqualene diphosphate + H+	Biosynthesis	0.03	0.03	0.03	0.03	No
R00703	(S)-Lactate + NAD+ = Pyruvate + NADH + H+	Central pathway	-101.40	-101.31	-66.45	-66.35	Yes
R00707_mt	L-Glutamate 5-semialdehyde_mt + NAD+_mt + H2O_mt = L-Glutamate_mt + NADH_mt + H+_mt	AA catabolism	1.39	3.20	1.29	3.26	No
R00709_mt	Isocitrate_mt + NAD+_mt = 2-Oxoglutarate_mt + CO2_mt + NADH_mt + H+_mt	Central pathway	4.98	6.79	5.77	7.45	No
R00716_mt	L-Lysine_mt + 2-Oxoglutarate_mt + NADPH_mt + H+_mt = N6-(L-1,3-Dicarboxypropyl)-L-lysine_mt + NADP+_mt + H2O_mt	AA catabolism	0.30	0.34	0.16	0.21	Yes
R00734	L-Tyrosine + 2-Oxoglutarate = 3-(4-Hydroxyphenyl)pyruvate + L-Glutamate	AA catabolism	0.30	0.34	0.34	0.39	Yes
R00742	ATP + Acetyl-CoA + HCO3- = ADP + Orthophosphate + Malonyl-CoA	Biosynthesis	3.67	5.07	3.67	5.21	No
R00782	L-Cysteine + H2O = Hydrogen sulfide + Pyruvate + NH3	AA catabolism	0.23	0.34	0.20	0.32	No
R00833_mt	(R)-2-Methyl-3-oxopropanoyl-CoA_mt = Succinyl-CoA_mt	AA catabolism	1.15	1.44	1.08	1.46	No
R00840	1L-myo-Inositol 1-phosphate = alpha-D-Glucose 6-phosphate	Biosynthesis	-0.02	-0.02	-0.02	-0.02	No
R00851	sn-Glycerol 3-phosphate + Acyl-CoA = 1-Acyl-sn-glycerol 3-phosphate + CoA	Biosynthesis	0.22	0.49	0.22	0.52	No
R00927_mt	CoA_mt + 2-Methylacetoacetyl-CoA_mt = Propanoyl-CoA_mt + Acetyl-CoA_mt	AA catabolism	0.58	0.62	0.51	0.55	Yes
R00935_mt	(S)-Methylmalonate semialdehyde_mt + CoA_mt + NAD+_mt = Propanoyl-CoA_mt + CO2_mt + NADH_mt + H+_mt	AA catabolism	0.36	0.41	0.37	0.43	No
R00939	Dihydrofolate + NADPH + H+ = Tetrahydrofolate + NADP+	Central pathway	0.03	0.03	0.03	0.03	No
R00945	5,10-Methylenetetrahydrofolate + Glycine + H2O = Tetrahydrofolate + L-Serine	Biosynthesis	-2.22	-0.31	-1.92	-0.38	No
R00946	5-Methyltetrahydrofolate + L-Homocysteine = Tetrahydrofolate + L-Methionine	Central pathway	0.26	0.39	0.27	0.41	No
R00959	alpha-D-Glucose 1-phosphate = alpha-D-Glucose 6-phosphate	Biosynthesis	-1.03	-0.34	-1.10	-0.34	No
R00965	Orotidine 5'-phosphate = UMP + CO2	Biosynthesis	0.21	0.21	0.21	0.21	No
R01001	L-Cystathionine + H2O = L-Cysteine + NH3 + 2-Oxobutanoate	AA catabolism	0.12	0.17	0.10	0.16	No
R01015	(2R)-2-Hydroxy-3-(phosphonooxy)-propanal = Glycerone phosphate	Central pathway	-59.35	-50.98	-42.36	-31.16	Yes
R01021	ATP + Choline = ADP + Choline phosphate	Biosynthesis	0.02	1.60	0.02	1.73	No
R01049	ATP + D-Ribose 5-phosphate = AMP + 5-Phospho-alpha-D-ribose 1-diphosphate	Biosynthesis	0.43	1.35	0.43	1.42	No
R01056	D-Ribose 5-phosphate = D-Ribulose 5-phosphate	Central pathway	-2.20	-0.25	-1.82	-0.25	No
R01061	(2R)-2-Hydroxy-3-(phosphonooxy)-propanal + Orthophosphate + NAD+ = 3-Phospho-D-glyceroyl phosphate + NADH + H+	Central pathway	103.29	107.50	63.58	69.21	Yes
R01070	beta-D-Fructose 1,6-bisphosphate = Glycerone phosphate + (2R)-2-Hydroxy-3-(phosphonooxy)-propanal	Central pathway	42.17	52.49	19.82	32.60	Yes
R01072	L-Glutamine + 5-Phospho-alpha-D-ribose 1-diphosphate + H2O = 5-Phosphoribosylamine + Pyrophosphate + L-Glutamate	Biosynthesis	0.22	0.38	0.22	0.38	No

Reaction ID	Reaction	Category	Flux at 2% oxygen (mmol/gDW) Lower & upper bound		Flux at 20% oxygen (mmol/gDW) Lower & upper bound		Significant difference
R01082_mt	(S)-Malate_mt = Fumarate_mt + H2O_mt	Central pathway	-12.20	-1.15	-17.74	-1.08	No
R01082	(S)-Malate = Fumarate + H2O	Central pathway	-12.53	-0.61	-18.59	-0.66	No
R01083	N6-(1,2-Dicarboxyethyl)-AMP = Fumarate + AMP	Biosynthesis	0.09	1.49	0.09	1.63	No
R01090_mt	L-Leucine_mt + 2-Oxoglutarate_mt = 4-Methyl-2-oxopentanoate_mt + L-Glutamate_mt	AA catabolism	0.56	0.60	0.49	0.53	Yes
R01121	ATP + (R)-5-Diphosphomevalonate = ADP + Orthophosphate + Isopentenyl diphosphate + CO2	Biosynthesis	0.21	0.21	0.21	0.21	No
R01123	Isopentenyl diphosphate = Dimethylallyl diphosphate	Biosynthesis	0.07	0.07	0.07	0.07	No
R01127	IMP + H2O = 1-(5'-Phosphoribosyl)-5-formamido-4-imidazolecarboxamide	Biosynthesis	-0.38	-0.22	-0.38	-0.22	No
R01135	GTP + IMP + L-Aspartate = GDP + Orthophosphate + N6-(1,2-Dicarboxyethyl)-AMP	Biosynthesis	0.09	1.49	0.09	1.63	No
R01137	ATP + dADP = ADP + dATP	Biosynthesis	0.03	1.43	0.03	1.57	No
R01168	L-Histidine = Urocanate + NH3	AA catabolism	0.04	0.10	0.04	0.11	No
R01185	1L-myo-Inositol 1-phosphate + H2O = myo-Inositol + Orthophosphate	AA catabolism	0.02	0.02	0.02	0.02	No
R01199_mt	Oxidized ferredoxin_mt + 2-Oxobutanoate_mt + CoA_mt = Reduced ferredoxin_mt + Propanoyl-CoA_mt + CO2_mt	AA catabolism	0.12	0.41	0.10	0.48	No
R01214_mt	L-Valine_mt + 2-Oxoglutarate_mt = 3-Methyl-2-oxobutanoic acid_mt + L-Glutamate_mt	AA catabolism	0.36	0.41	0.37	0.43	No
R01220	5,10-Methylenetetrahydrofolate + NADP+ = 5,10-Methenyltetrahydrofolate + NADPH + H+	Central pathway	0.35	4.17	0.33	3.40	No
R01221	Glycine + Tetrahydrofolate + NAD+ = 5,10-Methylenetetrahydrofolate + NH3 + CO2 + NADH + H+	AA catabolism	0.15	2.25	0.03	1.79	No
R01231	ATP + Xanthosine 5'-phosphate + L-Glutamine + H2O = AMP + Pyrophosphate + GMP + L-Glutamate	Biosynthesis	0.13	0.78	0.13	0.90	No
R01281	Palmitoyl-CoA + L-Serine = 3-Dehydrosphinganine + CoA + CO2	Biosynthesis	0.02	0.02	0.02	0.02	No
R01290	L-Serine + L-Homocysteine = L-Cystathionine + H2O	AA catabolism	0.12	0.17	0.10	0.16	No
R01320	S-Adenosyl-L-methionine + Phosphatidyl-N-dimethylethanolamine = S-Adenosyl-L-homocysteine + Phosphatidylcholine	Biosynthesis	0.14	0.15	0.14	0.15	No
R01360_mt	(S)-3-Hydroxy-3-methylglutaryl-CoA_mt = Acetyl-CoA_mt + Acetoacetate_mt	AA catabolism	0.56	2.00	0.49	2.07	No
R01364	4-Fumarylacetoacetate + H2O = Acetoacetate + Fumarate	AA catabolism	0.30	0.34	0.34	0.39	Yes
R01397	Carbamoyl phosphate + L-Aspartate = Orthophosphate + N-Carbamoyl-L-aspartate	Biosynthesis	0.21	0.21	0.21	0.21	No
R01456	Cholesta-5,7-dien-3beta-ol + NADPH + H+ = Cholesterol + NADP+	Biosynthesis	0.03	0.03	0.03	0.03	No
R01512	ATP + 3-Phospho-D-glycerate = ADP + 3-Phospho-D-glyceroyl phosphate	Central pathway	-107.51	-103.29	-69.21	-63.58	Yes
R01518	2-Phospho-D-glycerate = 3-Phospho-D-glycerate	Central pathway	-105.01	-103.29	-65.18	-63.58	Yes
R01624	Acetyl-CoA + Acyl-carrier protein = CoA + Acetyl-[acyl-carrier protein]	Biosynthesis	0.46	0.58	0.46	0.61	No
R01626	Malonyl-CoA + Acyl-carrier protein = CoA + Malonyl-[acyl-carrier protein]	Biosynthesis	3.67	4.37	3.67	4.59	No
R01655	5,10-Methenyltetrahydrofolate + H2O = 10-Formyltetrahydrofolate + H+	Central pathway	0.35	4.17	0.33	3.40	No
R01658	Dimethylallyl diphosphate + Isopentenyl diphosphate = Pyrophosphate + Geranyl diphosphate	Biosynthesis	0.07	0.07	0.07	0.07	No
R01786	ATP + alpha-D-Glucose = ADP + alpha-D-Glucose 6-phosphate	Central pathway	53.06	54.50	33.16	34.74	Yes
R01794	Dihydrobiopterin + NADPH + H+ = Tetrahydrobiopterin + NADP+	AA catabolism	0.15	0.19	0.16	0.21	No
R01795	Tetrahydrobiopterin + L-Phenylalanine + Oxygen = Dihydrobiopterin + L-Tyrosine + H2O	AA catabolism	0.15	0.19	0.16	0.21	No
R01799	CTP + Phosphatidate = Pyrophosphate + CDP-diacylglycerol	Biosynthesis	0.03	0.03	0.03	0.03	No
R01801	CDP-diacylglycerol + sn-Glycerol 3-phosphate = CMP + Phosphatidylglycerophosphate	Biosynthesis	0.01	0.01	0.01	0.01	No
R01802	CDP-diacylglycerol + myo-Inositol = CMP + 1-Phosphatidyl-D-myo-inositol	Biosynthesis	0.02	0.02	0.02	0.02	No

Reaction ID	Reaction	Category	Flux at 2% oxygen (mmol/gDW) Lower & upper bound		Flux at 20% oxygen (mmol/gDW) Lower & upper bound		Significant difference
R01857	ATP + dGDP = ADP + dGTP	Biosynthesis	0.02	1.42	0.02	1.56	No
R01859_mt	ATP_mt + Propanoyl-CoA_mt + HCO3-_mt = ADP_mt + Orthophosphate_mt + (S)-2-Methyl-3-oxopropanoyl-CoA_mt	AA catabolism	1.15	1.44	1.08	1.46	No
R01867	(S)-Dihydroorotate + Oxygen = Orotate + H2O2	AA catabolism	0.21	0.21	0.21	0.21	No
R01870	Orotate + 5-Phospho-alpha-D-ribose 1-diphosphate = Orotidine 5'-phosphate + Pyrophosphate	AA catabolism	0.21	0.21	0.21	0.21	No
R01890	CTP + Choline phosphate = Pyrophosphate + CDP-choline	Biosynthesis	0.02	0.90	0.02	0.97	No
R01891	CDP-choline + N-Acylsphingosine = CMP + Sphingomyelin	Biosynthesis	0.02	0.71	0.02	0.78	No
R01938	2-Aminomuconate + NADPH + H+ + H2O = 2-Oxoadipate + NH3 + NADP+	AA catabolism	0.04	0.06	0.06	0.08	No
R01939	L-2-Aminoadipate + 2-Oxoglutarate = 2-Oxoadipate + L-Glutamate	AA catabolism	0.30	0.34	0.16	0.21	Yes
R01959	L-Formylkynurenine + H2O = Formate + L-Kynurenine	AA catabolism	0.04	0.06	0.06	0.08	No
R01960	L-Kynurenine + Oxygen + NADPH + H+ = 3-Hydroxy-L-kynurenine + NADP+ + H2O	AA catabolism	0.04	0.06	0.06	0.08	No
R01978	Acetyl-CoA + H2O + Acetoacetyl-CoA = (S)-3-Hydroxy-3-methylglutaryl-CoA + CoA	Biosynthesis	0.21	0.21	0.21	0.21	No
R01993	(S)-Dihydroorotate + H2O = N-Carbamoyl-L-aspartate	Biosynthesis	-0.21	-0.21	-0.21	-0.21	No
R02003	Geranyl diphosphate + Isopentenyl diphosphate = Pyrophosphate + trans,trans-Farnesyl diphosphate	Biosynthesis	0.07	0.07	0.07	0.07	No
R02016	Oxidized thioredoxin + NADPH + H+ = Thioredoxin + NADP+	Biosynthesis	0.06	0.48	0.06	0.53	No
R02017	Thioredoxin + ADP = dADP + Oxidized thioredoxin + H2O	Biosynthesis	0.03	0.03	0.03	0.03	No
R02024	Thioredoxin + CDP = dCDP + Oxidized thioredoxin + H2O	Biosynthesis	0.02	0.35	0.02	0.39	No
R02029	Phosphatidylglycerophosphate + H2O = Phosphatidylglycerol + Orthophosphate	Biosynthesis	0.01	0.01	0.01	0.01	No
R02055	Phosphatidylserine = Phosphatidylethanolamine + CO2	Biosynthesis	0.19	0.19	0.19	0.19	No
R02056	S-Adenosyl-L-methionine + Phosphatidylethanolamine = S-Adenosyl-L-homocysteine + Phosphatidyl-N-methylethanolamine	Biosynthesis	0.14	0.15	0.14	0.15	No
R02082	(S)-3-Hydroxy-3-methylglutaryl-CoA + 2 NADPH + 2 H+ = (R)-Mevalonate + CoA + 2 NADP+	Biosynthesis	0.21	0.21	0.21	0.21	No
R02085_mt	(S)-3-Hydroxy-3-methylglutaryl-CoA_mt = 3-Methylglutaconyl-CoA_mt + H2O_mt	Biosynthesis	-0.60	-0.56	-0.53	-0.49	Yes
R02093	ATP + dTDP = ADP + dTTP	Biosynthesis	0.03	1.43	0.03	1.57	No
R02094	ATP + dTMP = ADP + dTDP	Biosynthesis	0.03	1.43	0.03	1.57	No
R02101	dUMP + 5,10-Methylenetetrahydrofolate = Dihydrofolate + dTMP	Biosynthesis	0.03	0.03	0.03	0.03	No
R02198_mt	L-Isoleucine_mt + 2-Oxoglutarate_mt = 3-Methyl-2-oxopentanoate_mt + L-Glutamate_mt	AA catabolism	0.58	0.62	0.51	0.55	Yes
R02239	Phosphatidate + H2O = 1,2-Diacyl-sn-glycerol + Orthophosphate	Biosynthesis	0.18	1.58	0.18	1.72	No
R02241	1-Acyl-sn-glycerol 3-phosphate + Acyl-CoA = Phosphatidate + CoA	Biosynthesis	0.22	0.68	0.22	0.73	No
R02245	ATP + (R)-Mevalonate = ADP + (R)-5-Phosphomevalonate	Biosynthesis	0.21	0.21	0.21	0.21	No
R02313_mt	N6-(L-1,3-Dicarboxypropyl)-L-lysine_mt + NAD+_mt + H2O_mt = L-Glutamate_mt + L-2-Aminoadipate 6-semialdehyde_mt + NADH_mt + H+_mt	AA catabolism	0.30	0.34	0.16	0.21	Yes
R02326	ATP + dCDP = ADP + dCTP	Biosynthesis	0.02	1.42	0.02	1.56	No
R02519	Homogentisate + Oxygen = 4-Maleylacetoacetate	AA catabolism	0.30	0.34	0.34	0.39	Yes
R02521	3-(4-Hydroxyphenyl)pyruvate + Oxygen = Homogentisate + CO2	AA catabolism	0.30	0.34	0.34	0.39	Yes
R02661_mt	2-Methylpropanoyl-CoA_mt + FAD_mt = 2-Methylprop-2-enoyl-CoA_mt + FADH2_mt	AA catabolism	0.36	0.41	0.37	0.43	No
R02662_mt	3-Methyl-2-oxobutanoic acid_mt + CoA_mt + NAD+_mt = 2-Methylpropanoyl-CoA_mt + CO2_mt + NADH_mt + H+_mt	AA catabolism	0.36	0.41	0.37	0.43	No

Reaction ID	Reaction	Category	Flux at 2% oxygen (mmol/gDW) Lower & upper bound		Flux at 20% oxygen (mmol/gDW) Lower & upper bound		Significant difference
R02665	3-Hydroxyanthranilate + Oxygen = 2-Amino-3-carboxymuconate semialdehyde	AA catabolism	0.04	0.06	0.06	0.08	No
R02668	3-Hydroxy-L-kynurenine + H2O = 3-Hydroxyanthranilate + L-Alanine	AA catabolism	0.04	0.06	0.06	0.08	No
R02765_mt	(R)-2-Methyl-3-oxopropanoyl-CoA_mt = (S)-2-Methyl-3-oxopropanoyl-CoA_mt	AA catabolism	-1.44	-1.15	-1.46	-1.08	No
R02814	Oleoyl-[acyl-carrier protein] + H2O = Acyl-carrier protein + (9Z)-Octadecenoic acid	Biosynthesis	0.45	0.52	0.45	0.53	No
R02872	Presqualene diphosphate + NADPH + H+ = Pyrophosphate + Squalene + NADP+	Biosynthesis	0.03	0.03	0.03	0.03	No
R02874	Squalene + Oxygen + NADPH + H+ = (S)-2,3-Epoxysqualene + NADP+ + H2O	Biosynthesis	0.03	0.03	0.03	0.03	No
R02914	Urocanate + H2O = 4-Imidazolone-5-propanoate	AA catabolism	0.04	0.10	0.04	0.11	No
R02978	3-Dehydrosphinganine + NADPH + H+ = Sphinganine + NADP+	Biosynthesis	0.02	0.02	0.02	0.02	No
R03103	L-2-Aminoadipate 6-semialdehyde + NADP+ + H2O = L-2-Aminoadipate + NADPH + H+	AA catabolism	0.30	1.04	0.16	0.98	No
R03172_mt	(S)-2-Methylbutanoyl-CoA_mt + FAD_mt = 2-Methylbut-2-enoyl-CoA_mt + FADH2_mt	AA catabolism	0.58	0.62	0.51	0.55	Yes
R03174_mt	3-Methyl-2-oxopentanoate_mt + CoA_mt + NAD+_mt = (S)-2-Methylbutanoyl-CoA_mt + CO2_mt + NADH_mt + H+_mt	AA catabolism	0.58	0.62	0.51	0.55	Yes
R03181	4-Maleylacetoacetate = 4-Fumarylacetoacetate	AA catabolism	0.30	0.34	0.34	0.39	Yes
R03199	(S)-2,3-Epoxysqualene = Lanosterol	Biosynthesis	0.03	0.03	0.03	0.03	No
R03245	ATP + (R)-5-Phosphomevalonate = ADP + (R)-5-Diphosphomevalonate	Biosynthesis	0.21	0.21	0.21	0.21	No
R03370	Octadecanoyl-[acyl-carrier protein] + Reduced acceptor + Oxygen = Oleoyl-[acyl-carrier protein] + Acceptor + 2 H2O	Biosynthesis	0.45	0.52	0.45	0.53	No
R03424	S-Adenosyl-L-methionine + Phosphatidyl-N-methylethanolamine = S-Adenosyl-L-homocysteine + Phosphatidyl-N-dimethylethanolamine	Biosynthesis	0.14	0.15	0.14	0.15	No
R03889	2-Aminomuconate semialdehyde + NAD+ + H2O = 2-Aminomuconate + H+ + NADH	AA catabolism	0.04	0.06	0.06	0.08	No
R04095_mt	3-Methylbutanoyl-CoA_mt + FAD_mt = 3-Methylcrotonyl-CoA_mt + FADH2_mt	AA catabolism	0.56	0.60	0.49	0.53	Yes
R04097_mt	4-Methyl-2-oxopentanoate_mt + CoA_mt + NAD+_mt = 3-Methylbutanoyl-CoA_mt + CO2_mt + NADH_mt + H+_mt	AA catabolism	0.56	0.60	0.49	0.53	Yes
R04138_mt	ATP_mt + 3-Methylcrotonyl-CoA_mt + HCO3-_mt = ADP_mt + Orthophosphate_mt + 3-Methylglutaconyl-CoA_mt	AA catabolism	0.56	0.60	0.49	0.53	Yes
R04144	ATP + 5-Phosphoribosylamine + Glycine = ADP + Orthophosphate + 5'-Phosphoribosylglycinamide	Biosynthesis	0.22	0.38	0.22	0.38	No
R04203_mt	(2S,3S)-3-Hydroxy-2-methylbutanoyl-CoA_mt + NAD+_mt = 2-Methylacetoacetyl-CoA_mt + NADH_mt + H+_mt	AA catabolism	0.58	0.62	0.51	0.55	Yes
R04204_mt	2-Methylbut-2-enoyl-CoA_mt + H2O_mt = (2S,3S)-3-Hydroxy-2-methylbutanoyl-CoA_mt	AA catabolism	0.58	0.62	0.51	0.55	Yes
R04208	ATP + 2-(Formamido)-N1-(5'-phosphoribosyl)acetamidine = ADP + Orthophosphate + Aminoimidazole ribotide	Biosynthesis	0.22	0.38	0.22	0.38	No
R04209	1-(5-Phospho-D-ribosyl)-5-amino-4-imidazolecarboxylate = Aminoimidazole ribotide + CO2	Biosynthesis	-0.38	-0.22	-0.38	-0.22	No
R04224_mt	2-Methylprop-2-enoyl-CoA_mt + H2O_mt = (S)-3-Hydroxyisobutyryl-CoA_mt	AA catabolism	0.36	0.41	0.37	0.43	No
R04323	2-Amino-3-carboxymuconate semialdehyde = 2-Aminomuconate semialdehyde + CO2	AA catabolism	0.04	0.06	0.06	0.08	No
R04325	10-Formyltetrahydrofolate + 5'-Phosphoribosylglycinamide = Tetrahydrofolate + 5'-Phosphoribosyl-N-formylglycinamide	Biosynthesis	0.22	0.38	0.22	0.38	No
R04355	Acetyl-[acyl-carrier protein] + Malonyl-[acyl-carrier protein] = Acetoacetyl-[acp] + CO2 + Acyl-carrier protein	Biosynthesis	0.46	0.58	0.46	0.61	No
R04428	(3R)-3-Hydroxybutanoyl-[acyl-carrier protein] = But-2-enoyl-[acyl-carrier protein] + H2O	Biosynthesis	0.46	0.58	0.46	0.61	No

Reaction ID	Reaction	Category	Flux at 2% oxygen (mmol/gDW) Lower & upper bound		Flux at 20% oxygen (mmol/gDW) Lower & upper bound		Significant difference
R04430	But-2-enoyl-[acyl-carrier protein] + NADPH + H+ = Butyryl-[acp] + NADP+	Biosynthesis	0.46	0.58	0.46	0.61	No
R04463	ATP + 5'-Phosphoribosyl-N-formylglycinamide + L-Glutamine + H2O = ADP + Orthophosphate + 2-(Formamido)-N1-(5'-phosphoribosyl)acetamidine + L-Glutamate	Biosynthesis	0.22	0.38	0.22	0.38	No
R04533	Acetoacetyl-[acp] + NADPH + H+ = (3R)-3-Hydroxybutanoyl-[acyl-carrier protein] + NADP+	Biosynthesis	0.46	0.58	0.46	0.61	No
R04534	3-Oxodecanoyl-[acp] + NADPH = (3R)-3-Hydroxydecanoyl-[acyl-carrier protein] + NADP+	Biosynthesis	0.46	0.58	0.46	0.61	No
R04535	(3R)-3-Hydroxydecanoyl-[acyl-carrier protein] = trans-Dec-2-enoyl-[acp] + H2O	Biosynthesis	0.46	0.58	0.46	0.61	No
R04536	3-Oxooctanoyl-[acp] + NADPH + H+ = (3R)-3-Hydroxyoctanoyl-[acyl-carrier protein] + NADP+	Biosynthesis	0.46	0.58	0.46	0.61	No
R04537	(3R)-3-Hydroxyoctanoyl-[acyl-carrier protein] = trans-Oct-2-enoyl-[acp] + H2O	Biosynthesis	0.46	0.58	0.46	0.61	No
R04543	3-Oxohexadecanoyl-[acp] + NADPH + H+ = (3R)-3-Hydroxypalmitoyl-[acyl-carrier protein] + NADP+	Biosynthesis	0.46	0.55	0.46	0.57	No
R04544	(3R)-3-Hydroxypalmitoyl-[acyl-carrier protein] = trans-Hexadec-2-enoyl-[acp] + H2O	Biosynthesis	0.46	0.55	0.46	0.57	No
R04559	1-(5'-Phosphoribosyl)-5-amino-4-(N-succinocarboxamide)-imidazole = Fumarate + 1-(5'-Phosphoribosyl)-5-amino-4-imidazolecarboxamide	Biosynthesis	0.22	0.38	0.22	0.38	No
R04560	10-Formyltetrahydrofolate + 1-(5'-Phosphoribosyl)-5-amino-4-imidazolecarboxamide = Tetrahydrofolate + 1-(5'-Phosphoribosyl)-5-formamido-4-imidazolecarboxamide	Biosynthesis	0.22	0.38	0.22	0.38	No
R04566	3-Oxotetradecanoyl-[acp] + NADPH = (3R)-3-Hydroxytetradecanoyl-[acyl-carrier protein] + NADP+	Biosynthesis	0.46	0.58	0.46	0.61	No
R04568	(3R)-3-Hydroxytetradecanoyl-[acyl-carrier protein] = trans-Tetradec-2-enoyl-[acp] + H2O	Biosynthesis	0.46	0.58	0.46	0.61	No
R04591	ATP + 1-(5-Phospho-D-ribosyl)-5-amino-4-imidazolecarboxylate + L-Aspartate = ADP + Orthophosphate + 1-(5'-Phosphoribosyl)-5-amino-4-(N-succinocarboxamide)-imidazole	Biosynthesis	0.22	0.38	0.22	0.38	No
R04725	trans-Dodec-2-enoyl-[acp] + NADPH + H+ = Dodecanoyl-[acyl-carrier protein] + NADP+	Biosynthesis	0.46	0.58	0.46	0.61	No
R04726	Dodecanoyl-[acyl-carrier protein] + Malonyl-[acyl-carrier protein] = 3-Oxotetradecanoyl-[acp] + CO2 + Acyl-carrier protein	Biosynthesis	0.46	0.58	0.46	0.61	No
R04779	ATP + beta-D-Fructose 6-phosphate = ADP + beta-D-Fructose 1,6-bisphosphate	Central pathway	42.17	53.88	19.83	34.12	Yes
R04804	Zymosterol = 5alpha-Cholesta-7,24-dien-3beta-ol	Biosynthesis	0.03	0.03	0.03	0.03	No
R04952	Butyryl-[acp] + Malonyl-[acyl-carrier protein] = 3-Oxohexanoyl-[acp] + CO2 + Acyl-carrier protein	Biosynthesis	0.46	0.58	0.46	0.61	No
R04953	3-Oxohexanoyl-[acp] + NADPH = (R)-3-Hydroxyhexanoyl-[acp] + NADP+	Biosynthesis	0.46	0.58	0.46	0.61	No
R04954	(R)-3-Hydroxyhexanoyl-[acp] = trans-Hex-2-enoyl-[acp] + H2O	Biosynthesis	0.46	0.58	0.46	0.61	No
R04956	trans-Hex-2-enoyl-[acp] + NADPH = Hexanoyl-[acp] + NADP+	Biosynthesis	0.46	0.58	0.46	0.61	No
R04957	Hexanoyl-[acp] + Malonyl-[acyl-carrier protein] = 3-Oxooctanoyl-[acp] + CO2 + Acyl-carrier protein	Biosynthesis	0.46	0.58	0.46	0.61	No
R04959	trans-Oct-2-enoyl-[acp] + NADPH = Octanoyl-[acp] + NADP+	Biosynthesis	0.46	0.58	0.46	0.61	No
R04960	Octanoyl-[acp] + Malonyl-[acyl-carrier protein] = 3-Oxodecanoyl-[acp] + CO2 + Acyl-carrier protein	Biosynthesis	0.46	0.58	0.46	0.61	No
R04962	trans-Dec-2-enoyl-[acp] + NADPH = Decanoyl-[acp] + NADP+	Biosynthesis	0.46	0.58	0.46	0.61	No
R04963	Decanoyl-[acp] + Malonyl-[acyl-carrier protein] = 3-Oxododecanoyl-[acp] + CO2 + Acyl-carrier protein	Biosynthesis	0.46	0.58	0.46	0.61	No
R04964	3-Oxododecanoyl-[acp] + NADPH = (R)-3-Hydroxydodecanoyl-[acp] + NADP+	Biosynthesis	0.46	0.58	0.46	0.61	No
R04965	(R)-3-Hydroxydodecanoyl-[acp] = trans-Dodec-2-enoyl-[acp] + H2O	Biosynthesis	0.46	0.58	0.46	0.61	No

Reaction ID	Reaction	Category	Flux at 2% oxygen (mmol/gDW) Lower & upper bound		Flux at 20% oxygen (mmol/gDW) Lower & upper bound		Significant difference
R04967	trans-Tetradec-2-enoyl-[acp] + NADPH = Tetradecanoyl-[acp] + NADP+	Biosynthesis	0.46	0.58	0.46	0.61	No
R04968	Tetradecanoyl-[acp] + Malonyl-[acyl-carrier protein] = 3-Oxohexadecanoyl-[acp] + CO2 + Acyl-carrier protein	Biosynthesis	0.46	0.55	0.46	0.57	No
R04970	trans-Hexadec-2-enoyl-[acp] + NADPH = Hexadecanoyl-[acp] + NADP+	Biosynthesis	0.46	0.55	0.46	0.57	No
R05064_mt	(S)-3-Hydroxyisobutyryl-CoA_mt + H2O_mt = CoA_mt + (S)-3-Hydroxyisobutyrate_mt	AA catabolism	0.36	0.41	0.37	0.43	No
R05066_mt	(S)-3-Hydroxyisobutyrate_mt + NAD+_mt = (S)-Methylmalonate semialdehyde_mt + NADH_mt + H+_mt	AA catabolism	0.36	0.41	0.37	0.43	No
R05639	14-Demethyllanosterol + NADP+ = 4,4-Dimethyl-5alpha-cholesta-8,14,24-trien-3beta-ol + NADPH + H+	Biosynthesis	-0.03	-0.03	-0.03	-0.03	No
R05640	Lanosterol + 3 Oxygen + 3 NADPH + 3 H+ = 4,4-Dimethyl-5alpha-cholesta-8,14,24-trien-3beta-ol + Formate + 3 NADP+ + 4 H2O	Biosynthesis	0.03	0.03	0.03	0.03	No
R05703	5alpha-Cholesta-7,24-dien-3beta-ol + NADPH + H+ = 5alpha-Cholest-7-en-3beta-ol + NADP+	Biosynthesis	0.03	0.03	0.03	0.03	No
R05875_mt	Reduced ferredoxin_mt + NAD+_mt = Oxidized ferredoxin_mt + NADH_mt + H+_mt	Central pathway	0.12	0.41	0.10	0.48	No
R06517	Acyl-CoA + Sphinganine = CoA + Dihydroceramide	Biosynthesis	0.02	0.71	0.02	0.78	No
R06519	Dihydroceramide + Reduced acceptor + Oxygen = N-Acylsphingosine + Acceptor + 2 H2O	Biosynthesis	0.02	0.02	0.02	0.02	No
R07494	4alpha-Methylzymosterol-4-carboxylate + NADP+ = 3-Keto-4-methylzymosterol + NADPH + H+ + CO2	Biosynthesis	0.03	0.03	0.03	0.03	No
R07495	3-Keto-4-methylzymosterol + NADP+ = 4alpha-Methylzymosterol + NADPH + H+	Biosynthesis	0.03	0.03	0.03	0.03	No
R07496	4alpha-Methylzymosterol = Zymosterol	Biosynthesis	0.03	0.03	0.03	0.03	No
R07509	14-Demethyllanosterol + NADPH + H+ + CO2 = 4alpha-Methylzymosterol-4-carboxylate + NADP+ + H2O	Biosynthesis	0.03	0.03	0.03	0.03	No
EF0013	Glycine_ext = Glycine	Exchange	0.26	0.39	0.00	0.15	Yes
EF0016	L-Proline_ext = L-Proline	Exchange	-0.23	-0.18	-0.01	0.05	Yes
TF0007	L-Glutamate + L-Aspartate_mt = L-Glutamate_mt + L-Aspartate	Transport	1.95	27.10	-2.61	31.30	No
R00342	(S)-Malate + NAD+ = Oxaloacetate + NADH + H+	Central pathway	-21.71	-1.54	-24.86	3.28	No
R00355	L-Aspartate + 2-Oxoglutarate = Oxaloacetate + L-Glutamate	Central pathway	1.54	26.69	-3.28	30.63	No
R00355_mt	L-Aspartate_mt + 2-Oxoglutarate_mt = Oxaloacetate_mt + L-Glutamate_mt	Central pathway	-27.10	-1.95	-31.30	2.61	No
R00485	L-Asparagine + H2O = L-Aspartate + NH3	AA catabolism	0.04	0.81	0.00	0.77	No
EF0014	H+_ext = H+	Exchange	-5.85	13.64	1.44	24.76	No

Note: Reaction ID or component followed by '_mt' indicates that the reaction takes place or the reactant is located within the mitochondria.
